# Supplementary figures and images for: PDZ-directed substrate recruitment is the primary determinant of specific 4E-BP1 dephosphorylation by PP1-Neurabin
Source: eLife. 2025 Jun 23;13:RP103403. doi: 10.7554/eLife.103403 (PMC12185105; doi:10.7554/eLife.103403)

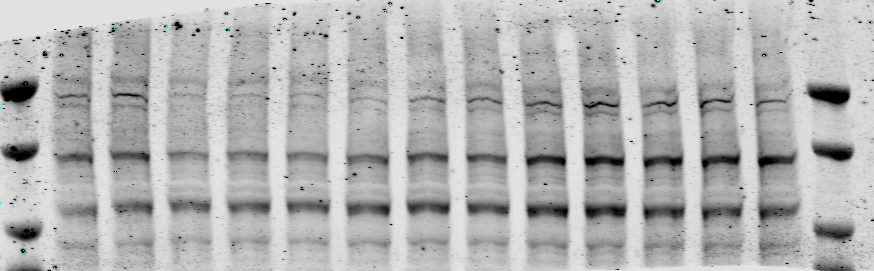

Supplement: Figure 1—source data 2. [file elife-103403-fig1-data2.zip › Figure 1B western blots/Afadin pS1275.png]

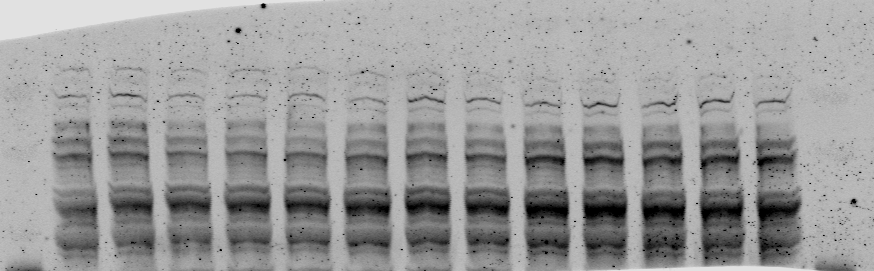

Supplement: Figure 1—source data 2. [file elife-103403-fig1-data2.zip › Figure 1B western blots/Afadin total.png]

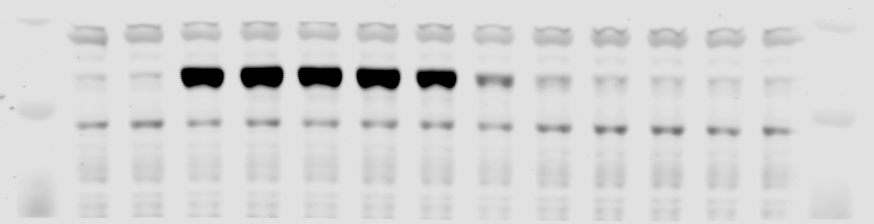

Supplement: Figure 1—source data 2. [file elife-103403-fig1-data2.zip › Figure 1B western blots/Flag fusion induction.png]

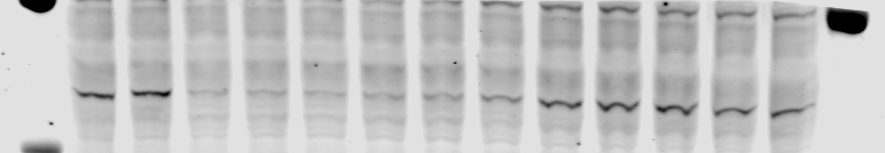

Supplement: Figure 1—source data 2. [file elife-103403-fig1-data2.zip › Figure 1B western blots/IRSp53 pS455.png]

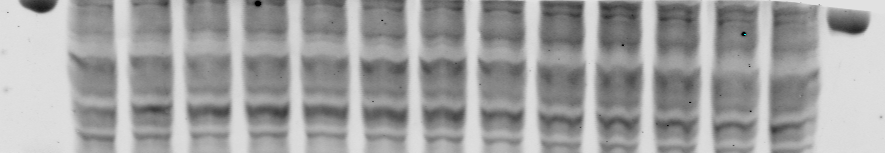

Supplement: Figure 1—source data 2. [file elife-103403-fig1-data2.zip › Figure 1B western blots/IRSp53 total.png]

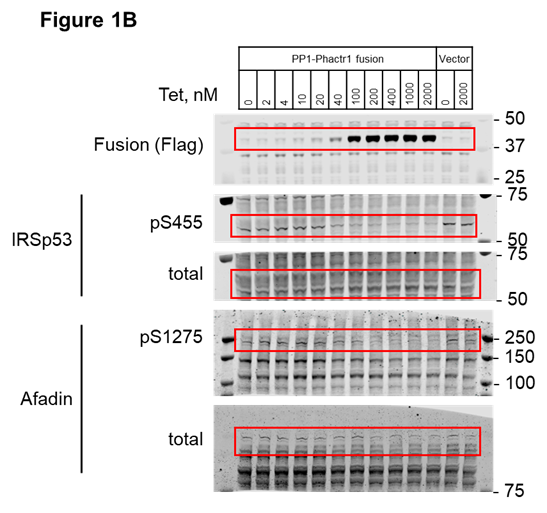

Supplement: Figure 1—source data 2. [file elife-103403-fig1-data2.zip › Figure 1B.png]

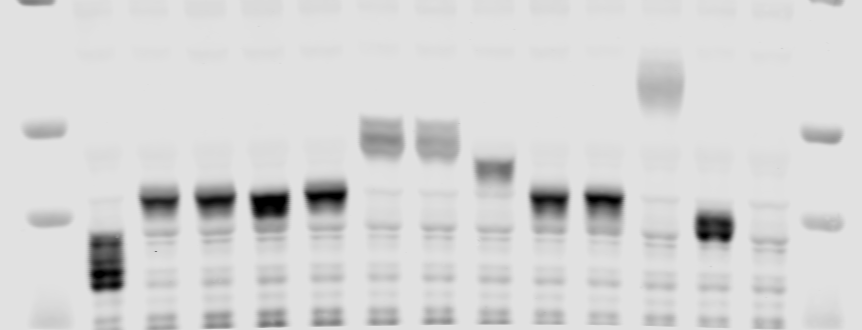

Supplement: Figure 1—source data 4. [file elife-103403-fig1-data4.zip › Figure 1C western blots/Flag-fusions.png]

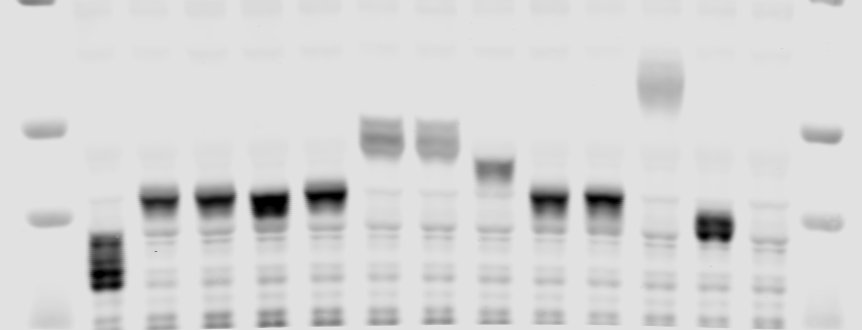

Supplement: Figure 1—source data 4. [file elife-103403-fig1-data4.zip › Figure 1C western blots/Flag-fusions.tif]

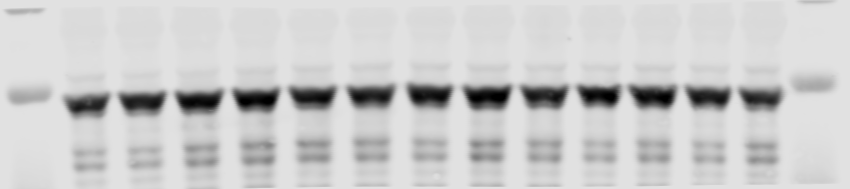

Supplement: Figure 1—source data 4. [file elife-103403-fig1-data4.zip › Figure 1C western blots/GAPDH.png]

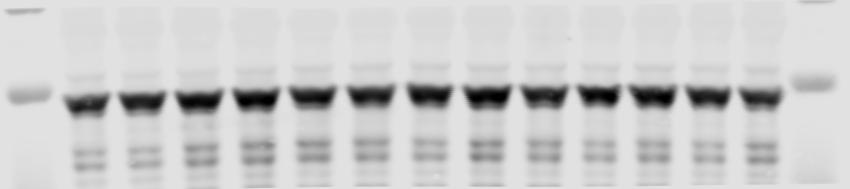

Supplement: Figure 1—source data 4. [file elife-103403-fig1-data4.zip › Figure 1C western blots/GAPDH.tif]

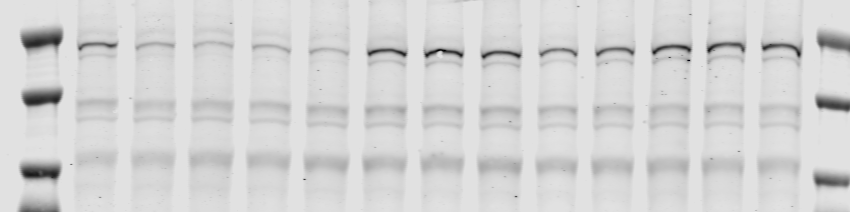

Supplement: Figure 1—source data 4. [file elife-103403-fig1-data4.zip › Figure 1C western blots/pAfadin.png]

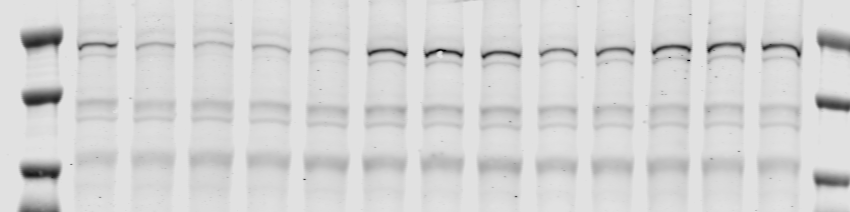

Supplement: Figure 1—source data 4. [file elife-103403-fig1-data4.zip › Figure 1C western blots/pAfadin.tif]

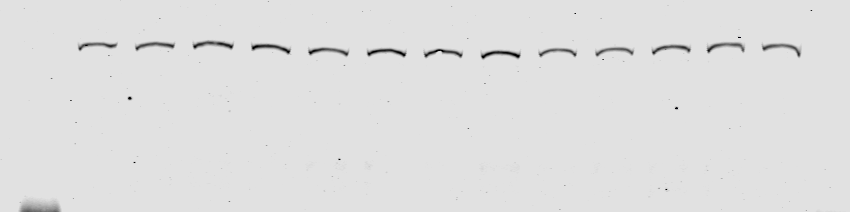

Supplement: Figure 1—source data 4. [file elife-103403-fig1-data4.zip › Figure 1C western blots/tAfadin.png]

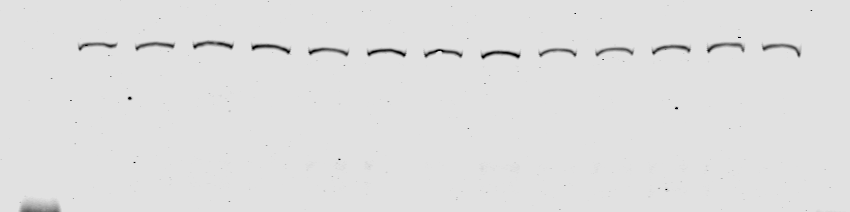

Supplement: Figure 1—source data 4. [file elife-103403-fig1-data4.zip › Figure 1C western blots/tAfadin.tif]

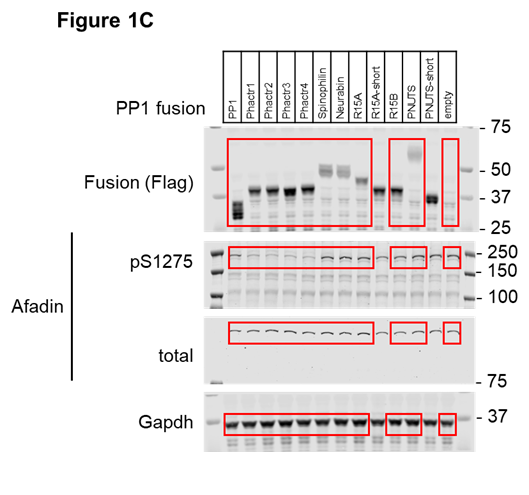

Supplement: Figure 1—source data 4. [file elife-103403-fig1-data4.zip › Figure 1C.png]

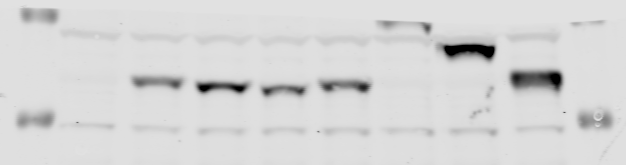

Supplement: Figure 1—figure supplement 1—source data 1. [file elife-103403-fig1-figsupp1-data1.zip › Figure S1B western blots/12Jun2020 flag.png]

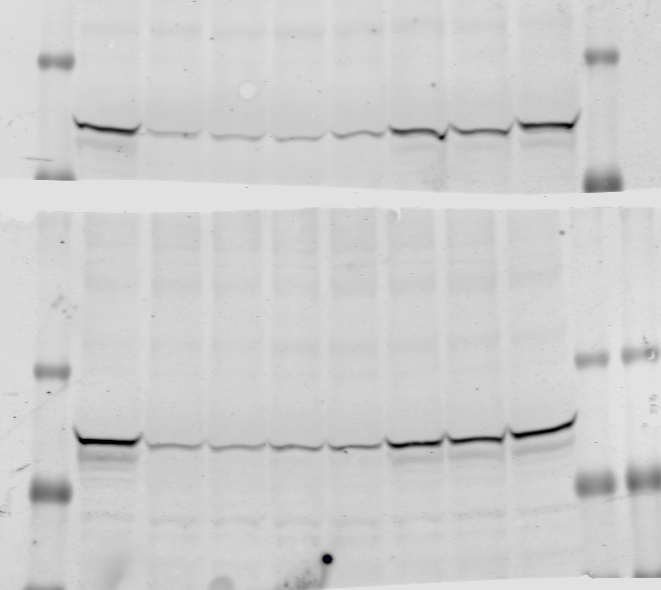

Supplement: Figure 1—figure supplement 1—source data 1. [file elife-103403-fig1-figsupp1-data1.zip › Figure S1B western blots/12Jun2020 pIRSp53.png]

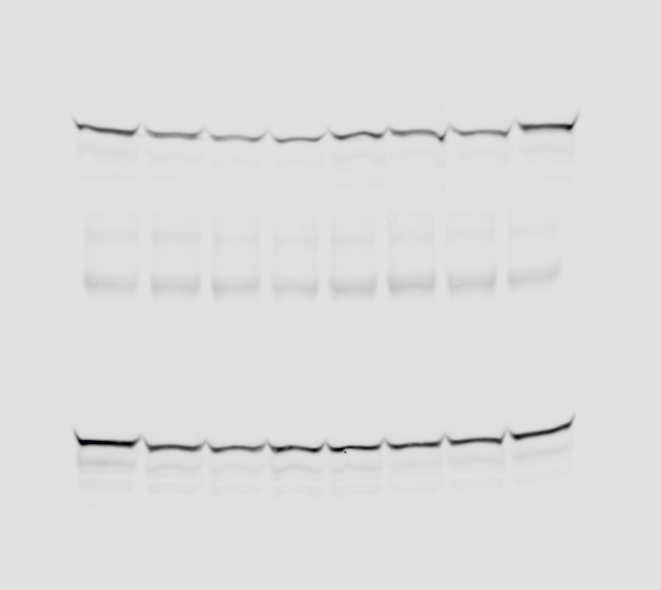

Supplement: Figure 1—figure supplement 1—source data 1. [file elife-103403-fig1-figsupp1-data1.zip › Figure S1B western blots/12Jun2020 tIRSp53.png]

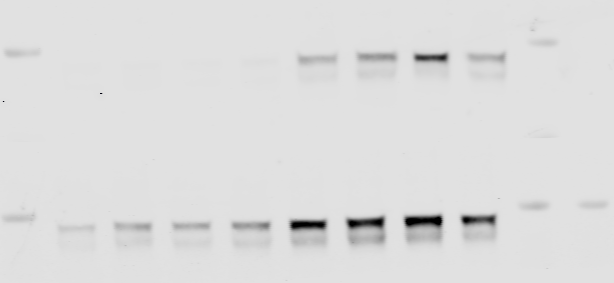

Supplement: Figure 1—figure supplement 1—source data 1. [file elife-103403-fig1-figsupp1-data1.zip › Figure S1B western blots/13Aug2020 flag.png]

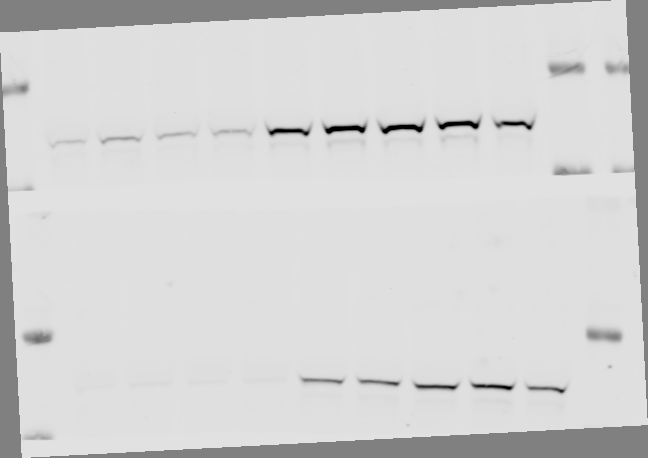

Supplement: Figure 1—figure supplement 1—source data 1. [file elife-103403-fig1-figsupp1-data1.zip › Figure S1B western blots/13Aug2020 pIRSp53.png]

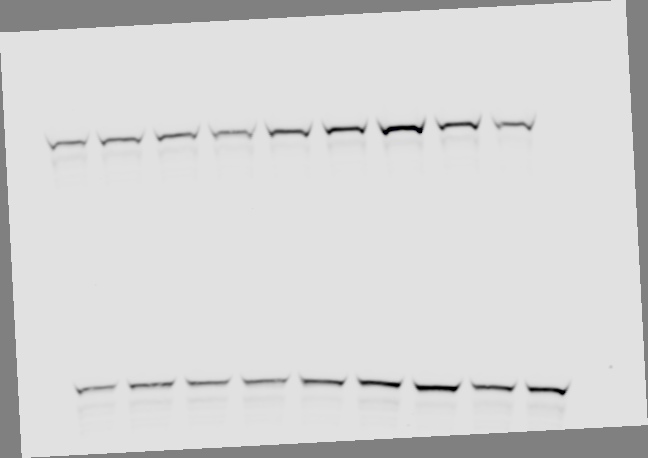

Supplement: Figure 1—figure supplement 1—source data 1. [file elife-103403-fig1-figsupp1-data1.zip › Figure S1B western blots/13Aug2020 tIRSp53.png]

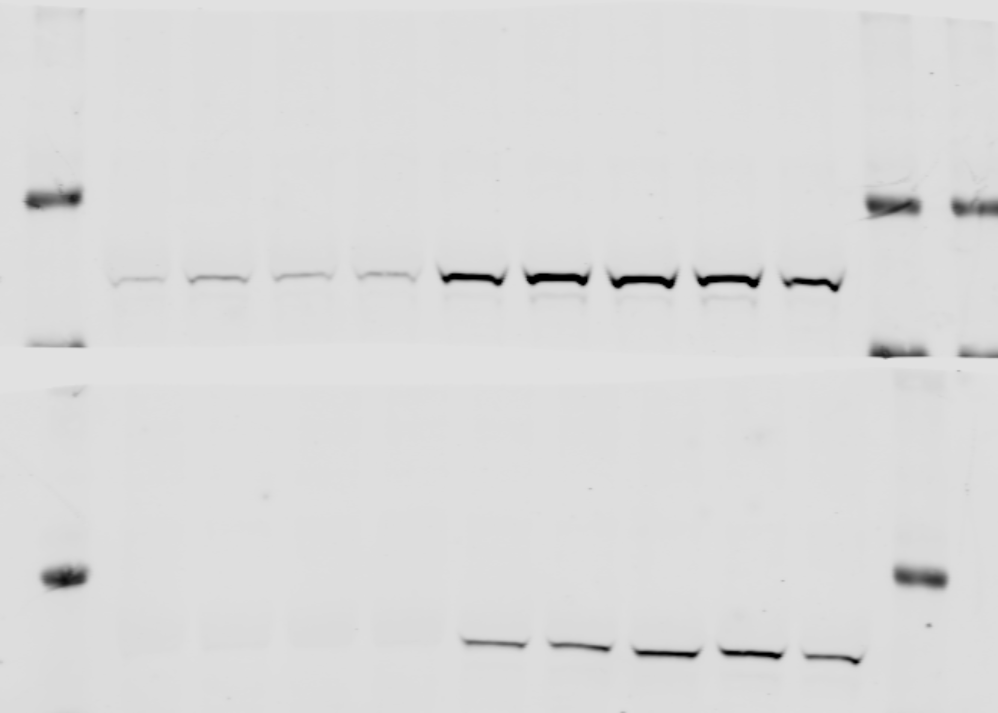

Supplement: Figure 1—figure supplement 1—source data 1. [file elife-103403-fig1-figsupp1-data1.zip › Figure S1B western blots/18Aug2020 pIRSp53.png]

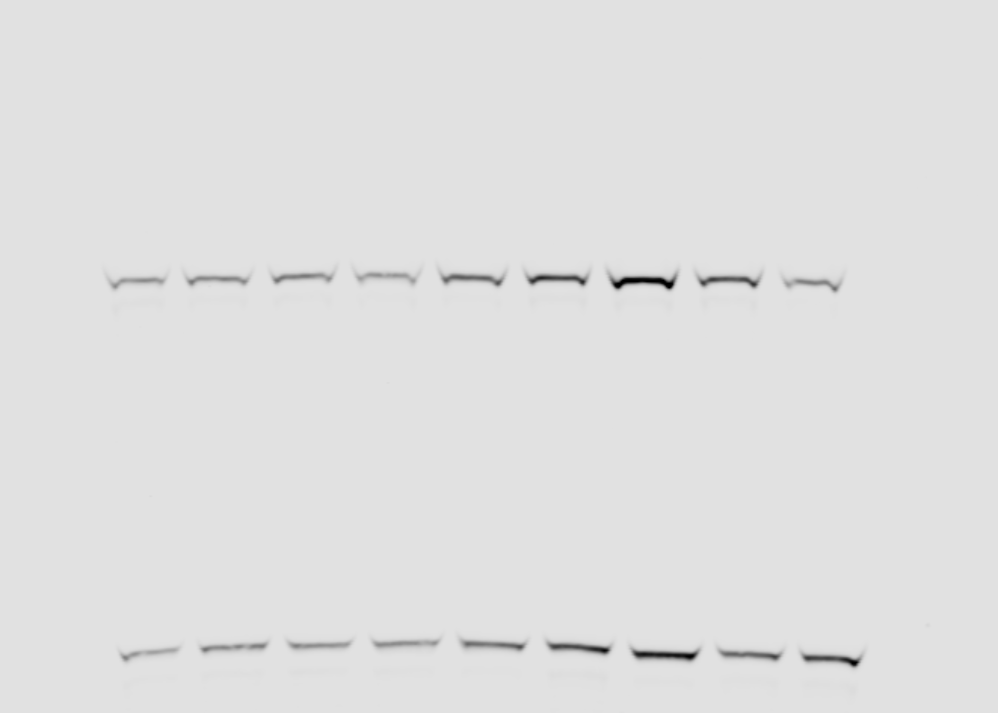

Supplement: Figure 1—figure supplement 1—source data 1. [file elife-103403-fig1-figsupp1-data1.zip › Figure S1B western blots/18Aug2020 tIRSp53.png]

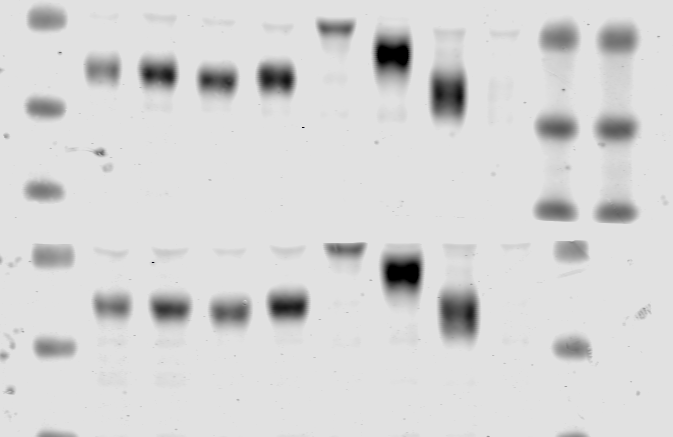

Supplement: Figure 1—figure supplement 1—source data 1. [file elife-103403-fig1-figsupp1-data1.zip › Figure S1B western blots/19Jun2020 flag.png]

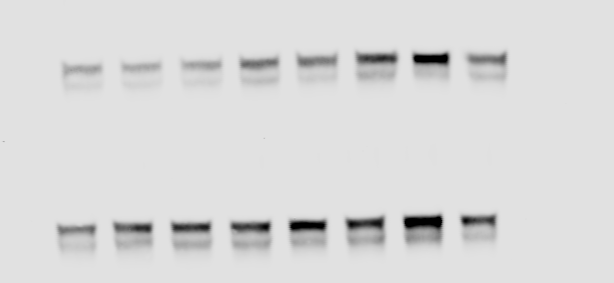

Supplement: Figure 1—figure supplement 1—source data 1. [file elife-103403-fig1-figsupp1-data1.zip › Figure S1B western blots/19Jun2020 tIRSp53.png]

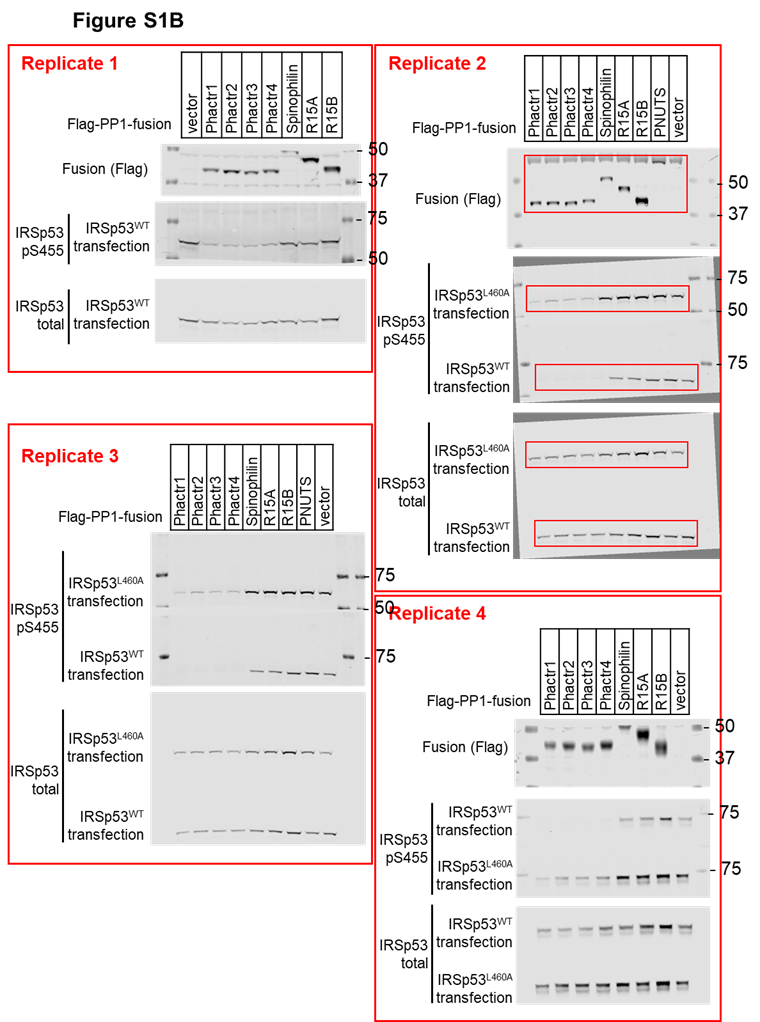

Supplement: Figure 1—figure supplement 1—source data 1. [file elife-103403-fig1-figsupp1-data1.zip › Figure S1B.png]

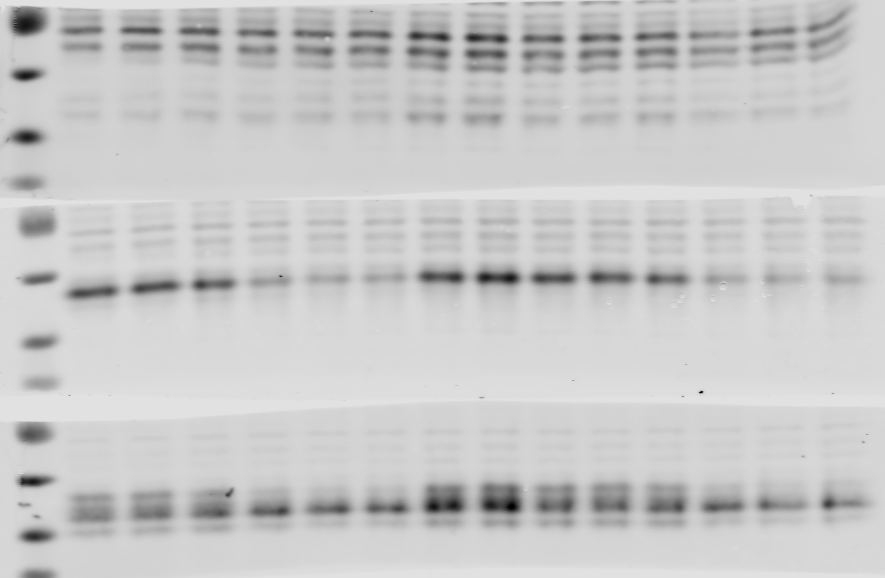

Supplement: Figure 3—source data 1. [file elife-103403-fig3-data1.zip › Figures_3D_and_S4C-western_blots/14Mar2024 4EBP1 p37-65-70.png]

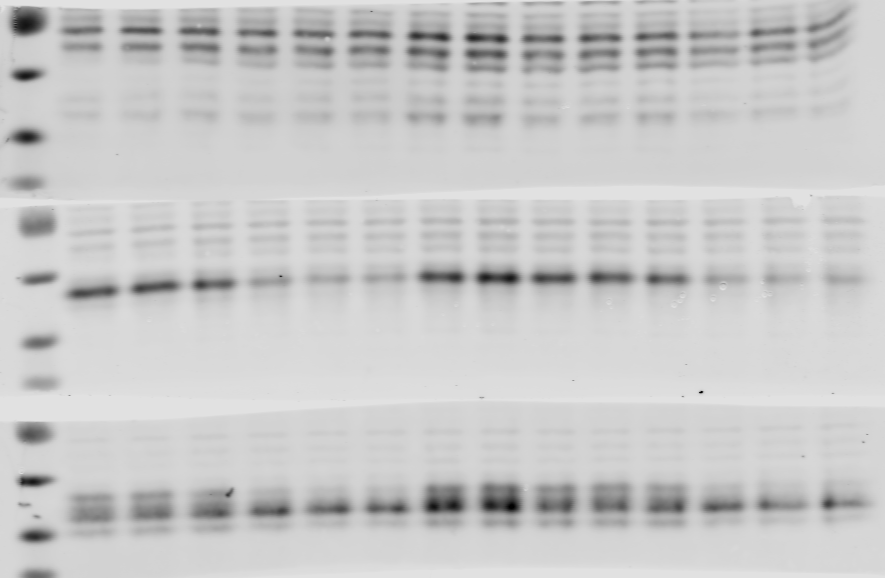

Supplement: Figure 3—source data 1. [file elife-103403-fig3-data1.zip › Figures_3D_and_S4C-western_blots/14Mar2024 4EBP1 p37-65-70.tif]

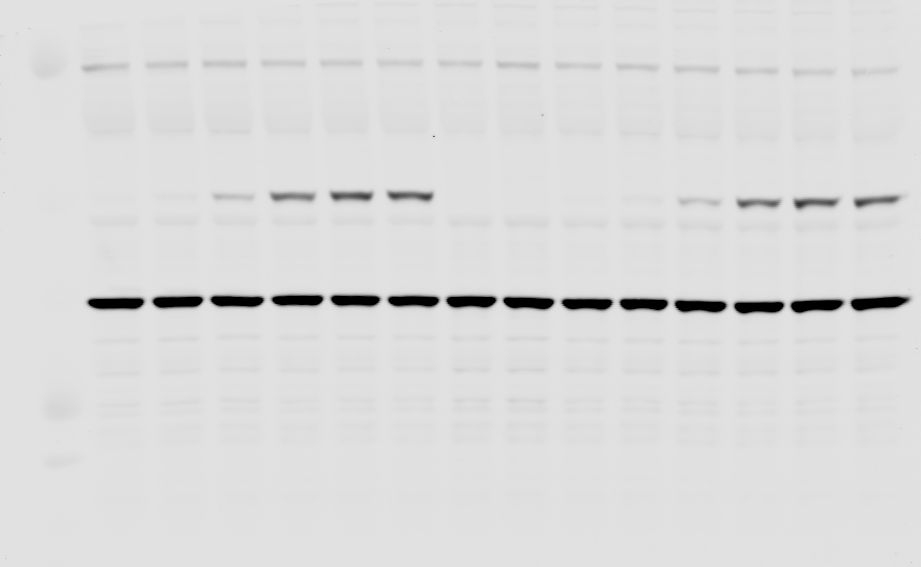

Supplement: Figure 3—source data 1. [file elife-103403-fig3-data1.zip › Figures_3D_and_S4C-western_blots/14Mar2024 flag-gapdh.png]

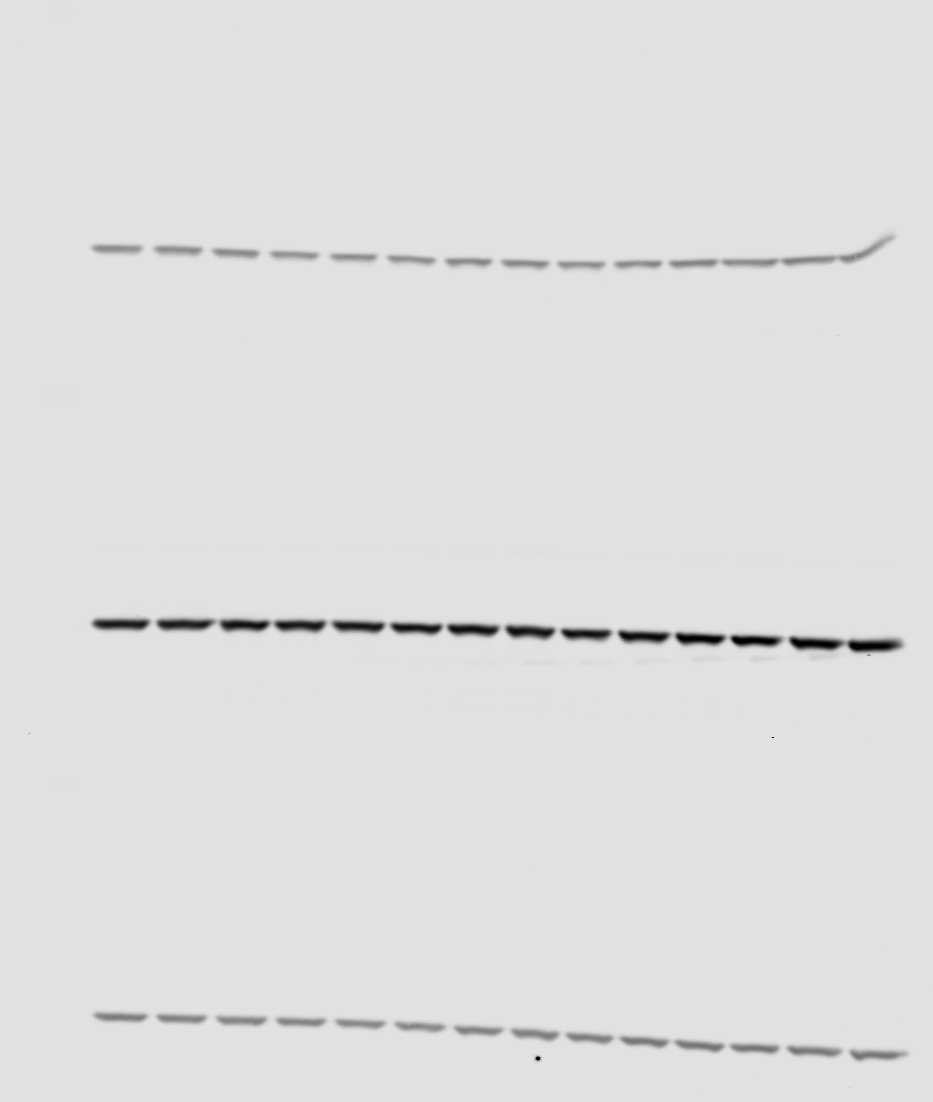

Supplement: Figure 3—source data 1. [file elife-103403-fig3-data1.zip › Figures_3D_and_S4C-western_blots/14Mar2024 gapdh.png]

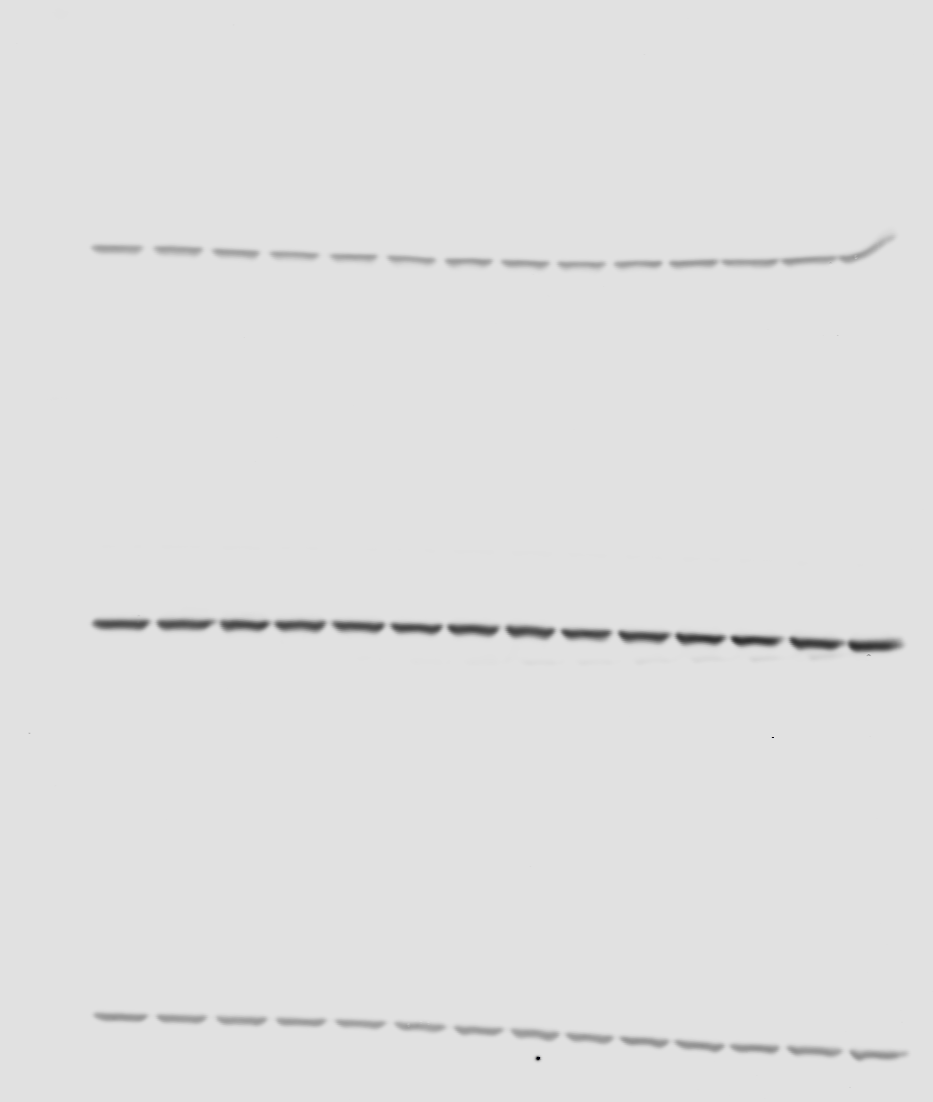

Supplement: Figure 3—source data 1. [file elife-103403-fig3-data1.zip › Figures_3D_and_S4C-western_blots/14Mar2024 gapdh.tif]

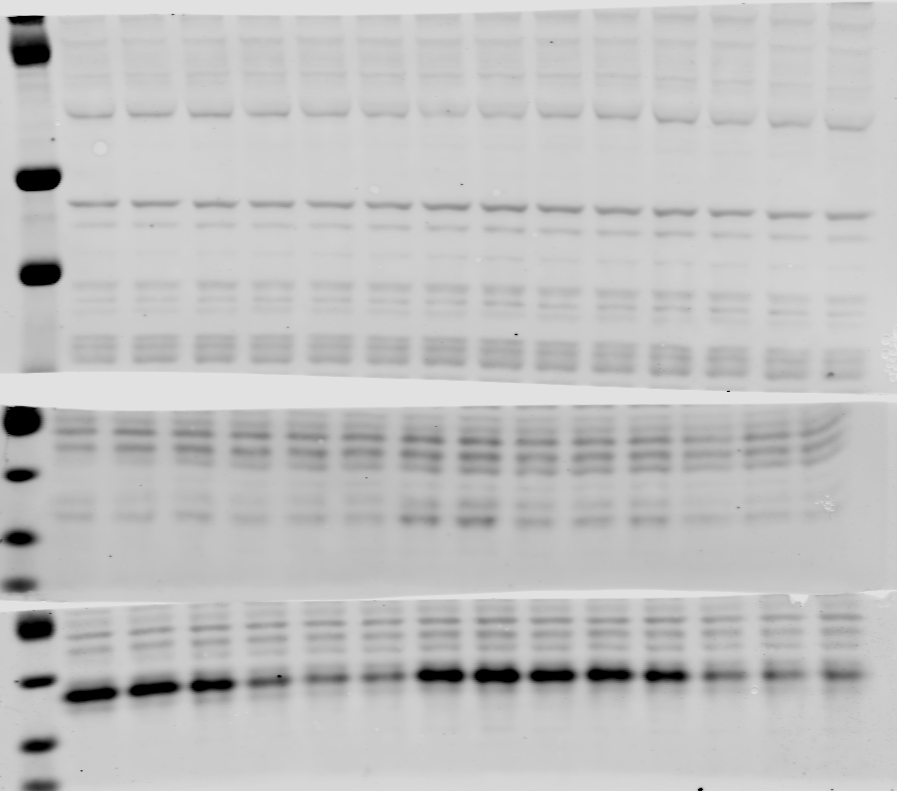

Supplement: Figure 3—source data 1. [file elife-103403-fig3-data1.zip › Figures_3D_and_S4C-western_blots/14Mar2024 repeat 4EBP1 p65-70-389.png]

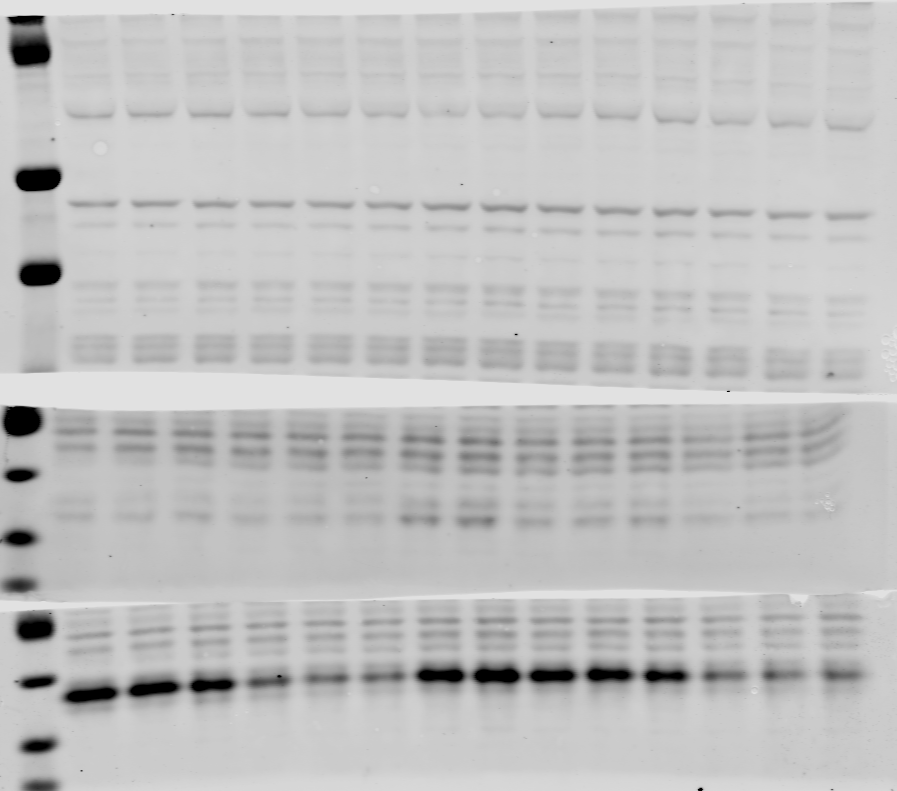

Supplement: Figure 3—source data 1. [file elife-103403-fig3-data1.zip › Figures_3D_and_S4C-western_blots/14Mar2024 repeat 4EBP1 p65-70-389.tif]

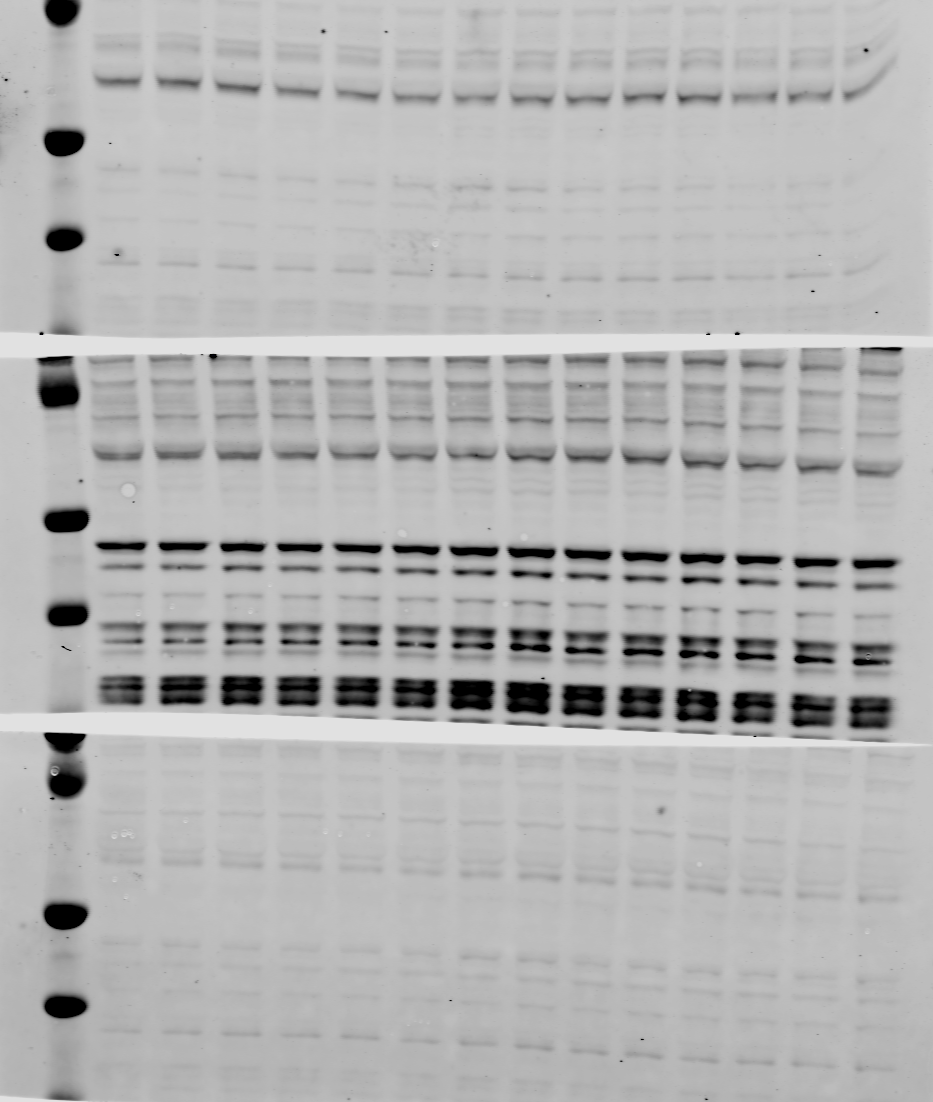

Supplement: Figure 3—source data 1. [file elife-103403-fig3-data1.zip › Figures_3D_and_S4C-western_blots/14Mar2024 S6K p371-389-421.png]

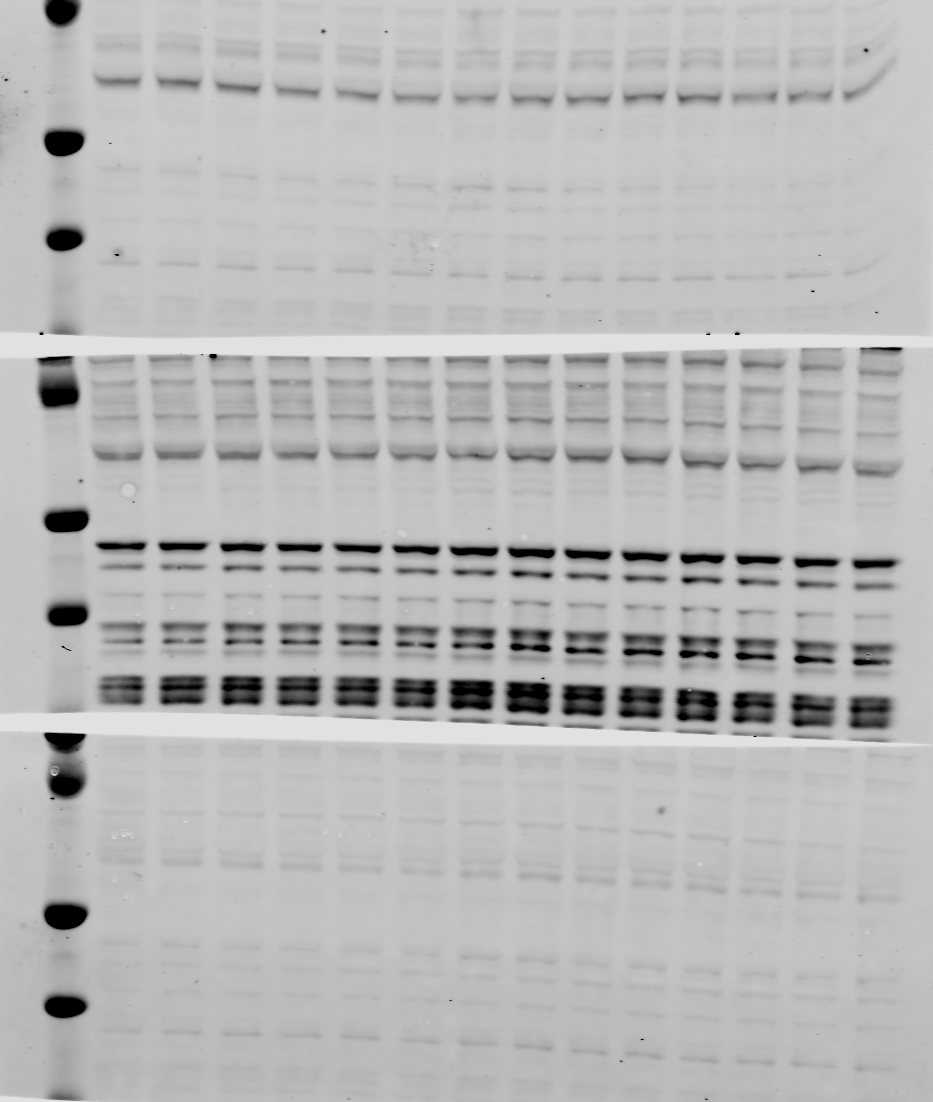

Supplement: Figure 3—source data 1. [file elife-103403-fig3-data1.zip › Figures_3D_and_S4C-western_blots/14Mar2024 S6K p371-389-421.tif]

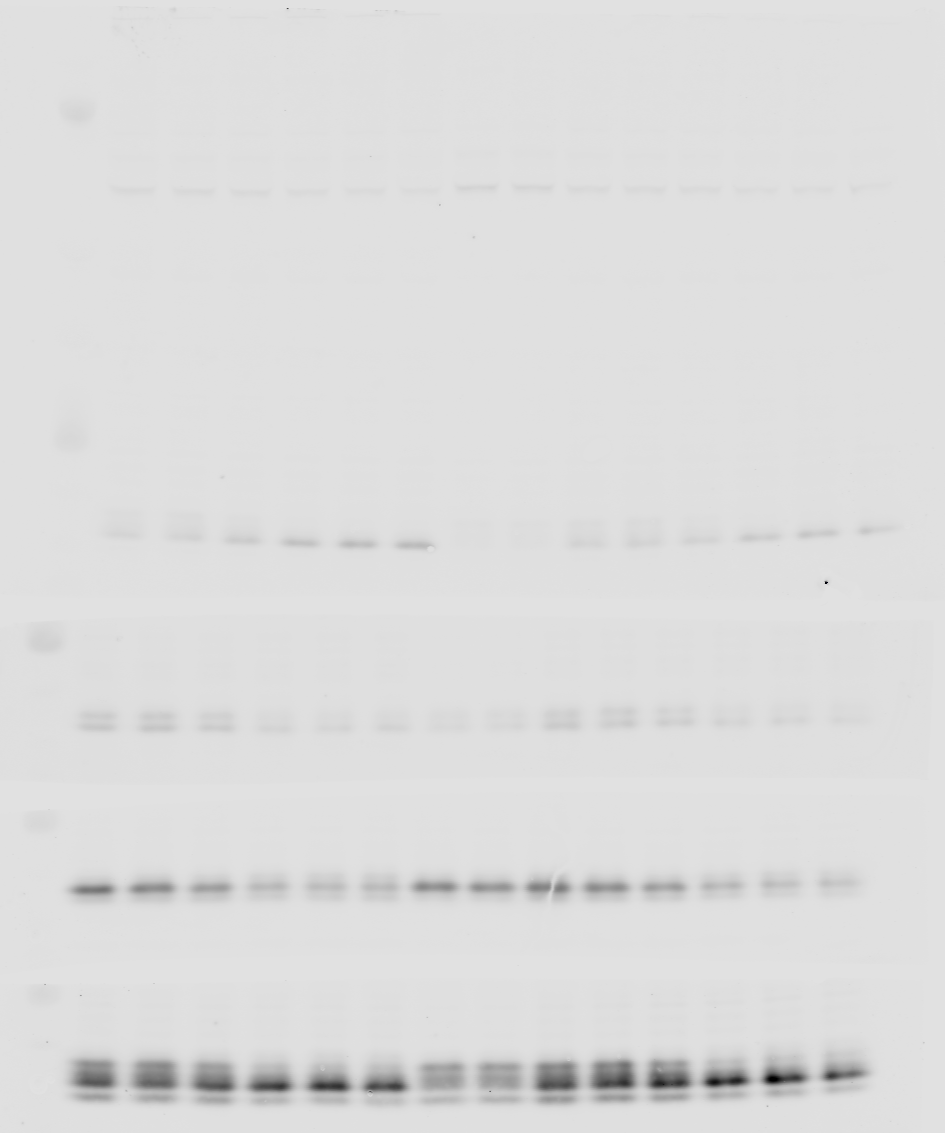

Supplement: Figure 3—source data 1. [file elife-103403-fig3-data1.zip › Figures_3D_and_S4C-western_blots/16Feb2024 4EBP1 p37-.png]

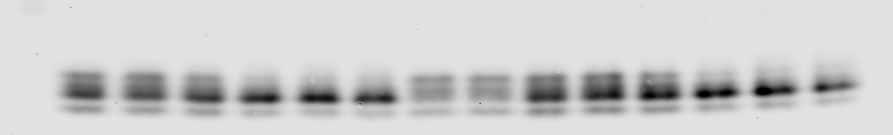

Supplement: Figure 3—source data 1. [file elife-103403-fig3-data1.zip › Figures_3D_and_S4C-western_blots/16Feb2024 4EBP1 p37.png]

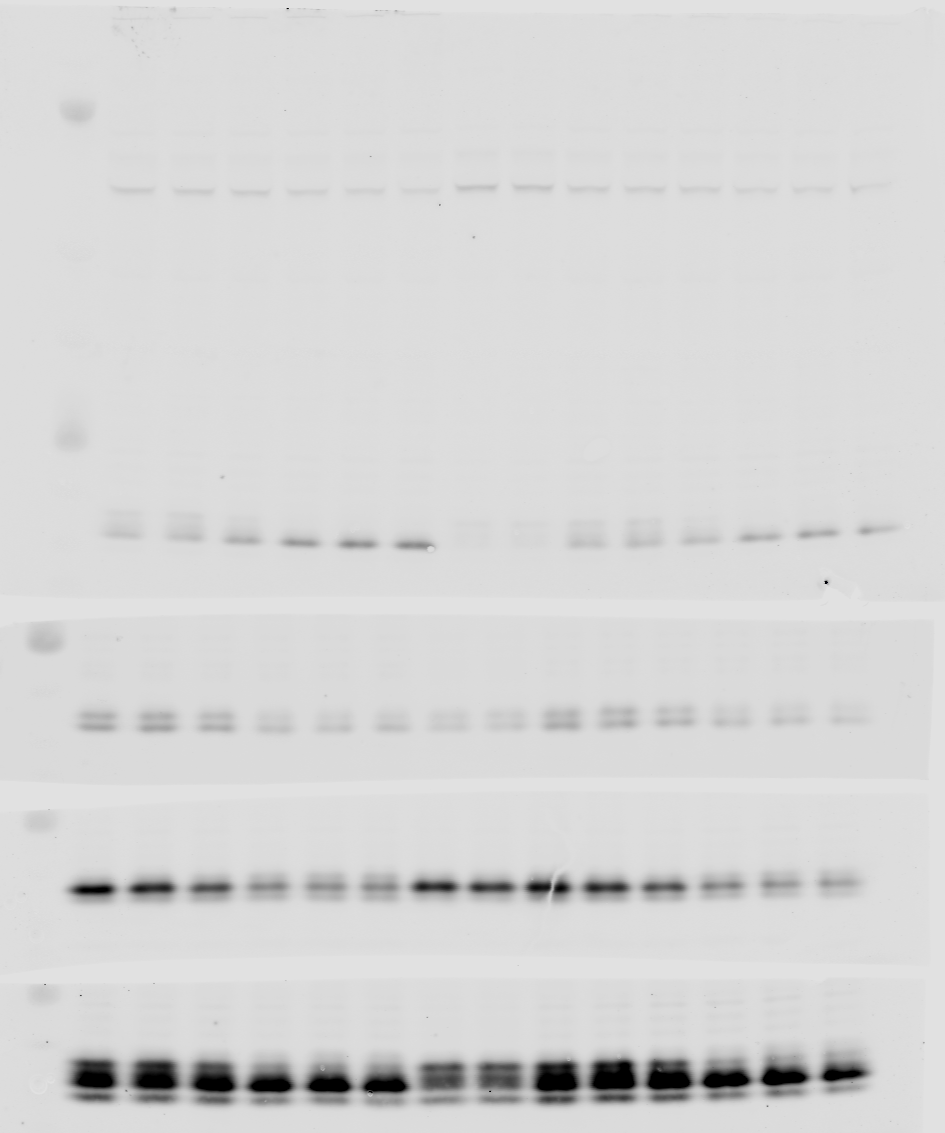

Supplement: Figure 3—source data 1. [file elife-103403-fig3-data1.zip › Figures_3D_and_S4C-western_blots/16Feb2024 4EBP1 p65-.png]

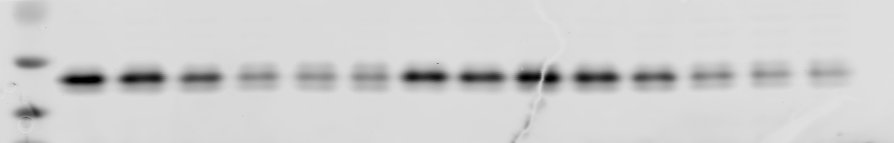

Supplement: Figure 3—source data 1. [file elife-103403-fig3-data1.zip › Figures_3D_and_S4C-western_blots/16Feb2024 4EBP1 p65.png]

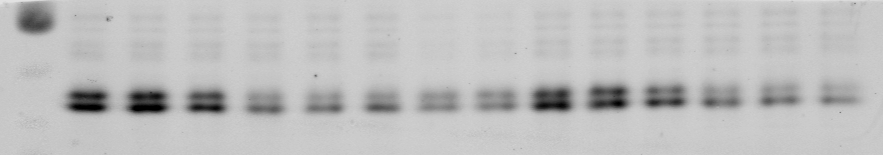

Supplement: Figure 3—source data 1. [file elife-103403-fig3-data1.zip › Figures_3D_and_S4C-western_blots/16Feb2024 4EBP1 p70.png]

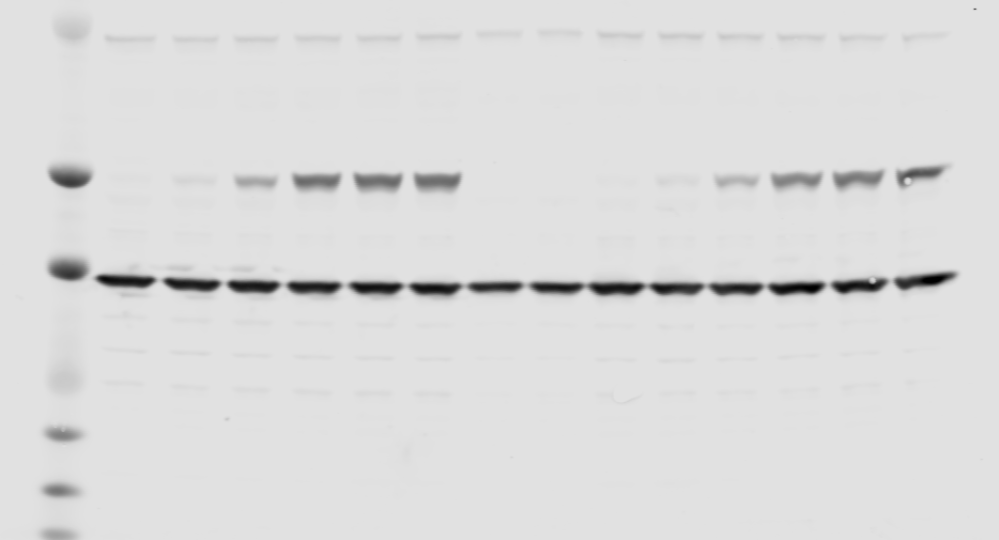

Supplement: Figure 3—source data 1. [file elife-103403-fig3-data1.zip › Figures_3D_and_S4C-western_blots/16Feb2024 FLAG-GAPDH.png]

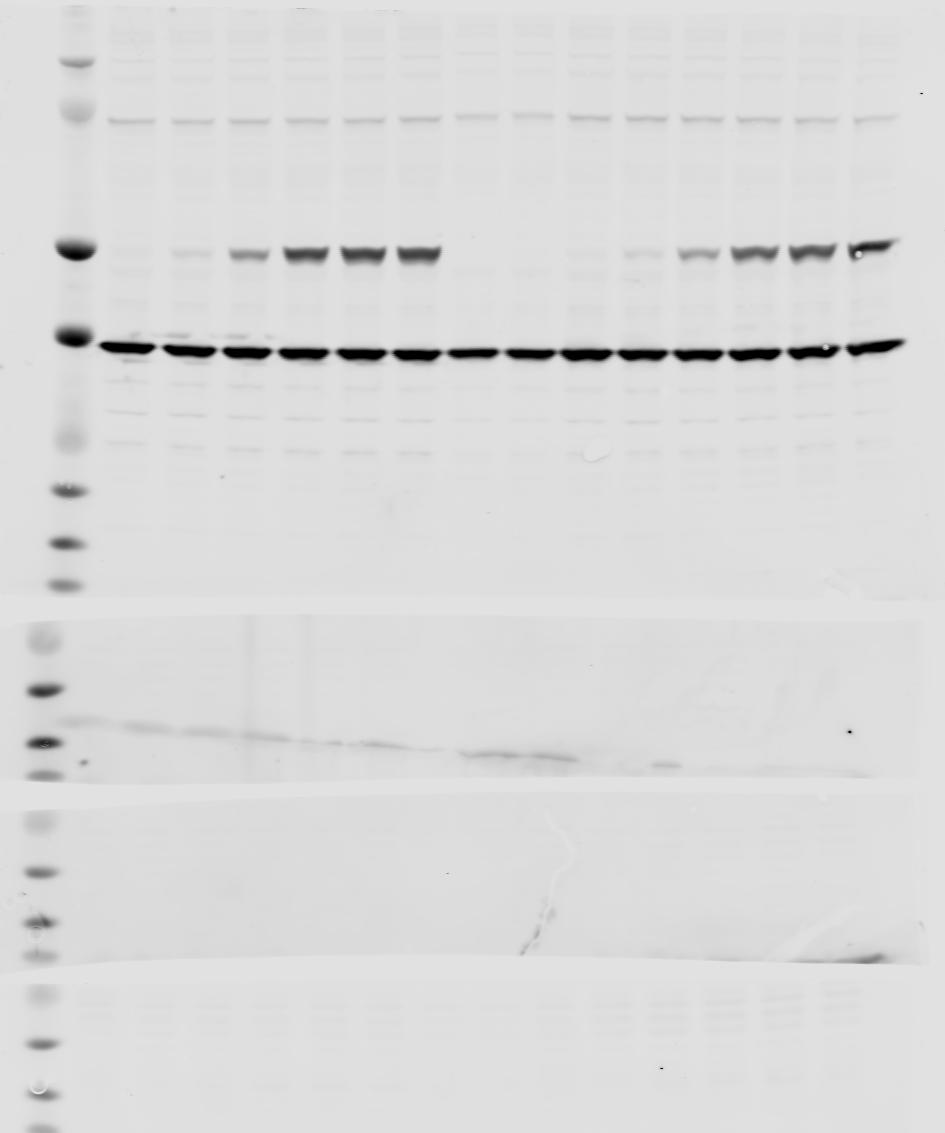

Supplement: Figure 3—source data 1. [file elife-103403-fig3-data1.zip › Figures_3D_and_S4C-western_blots/16Feb2024 FLAG-GAPDH.tif]

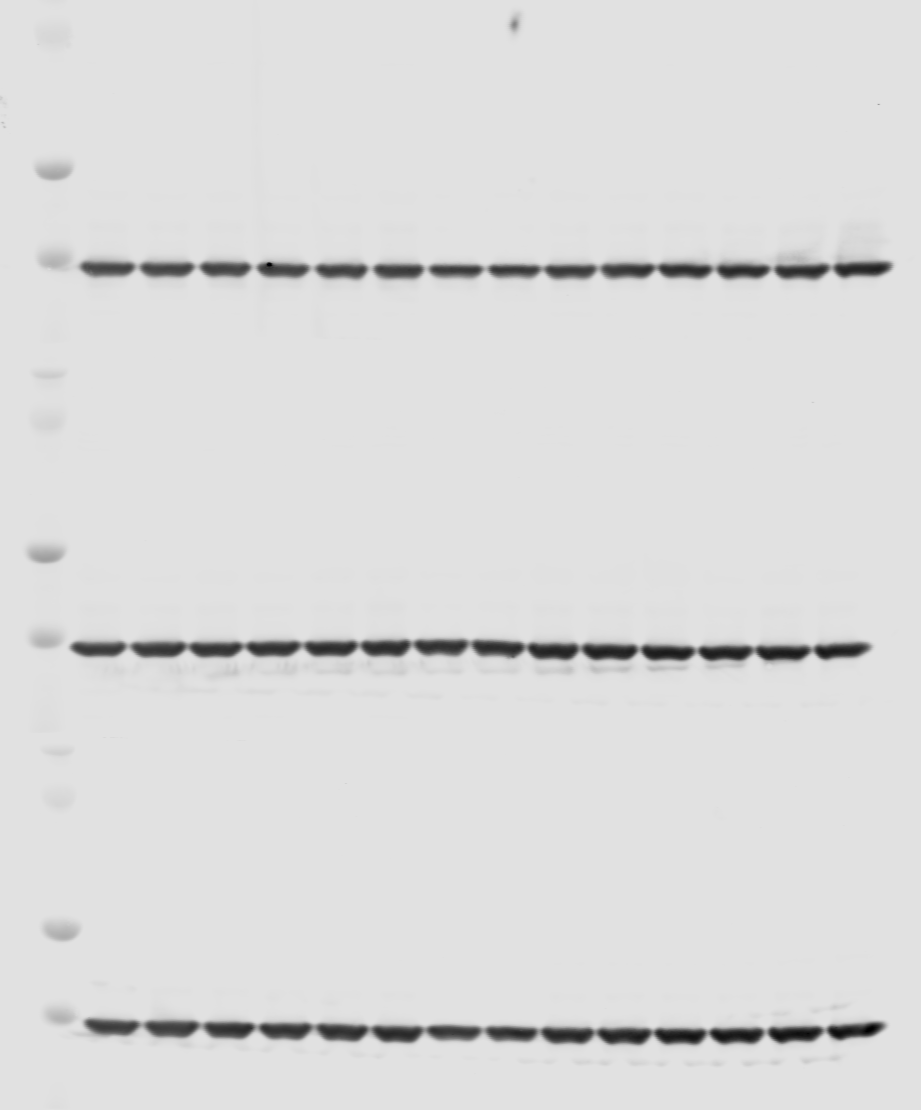

Supplement: Figure 3—source data 1. [file elife-103403-fig3-data1.zip › Figures_3D_and_S4C-western_blots/16Feb2024 GAPDH.png]

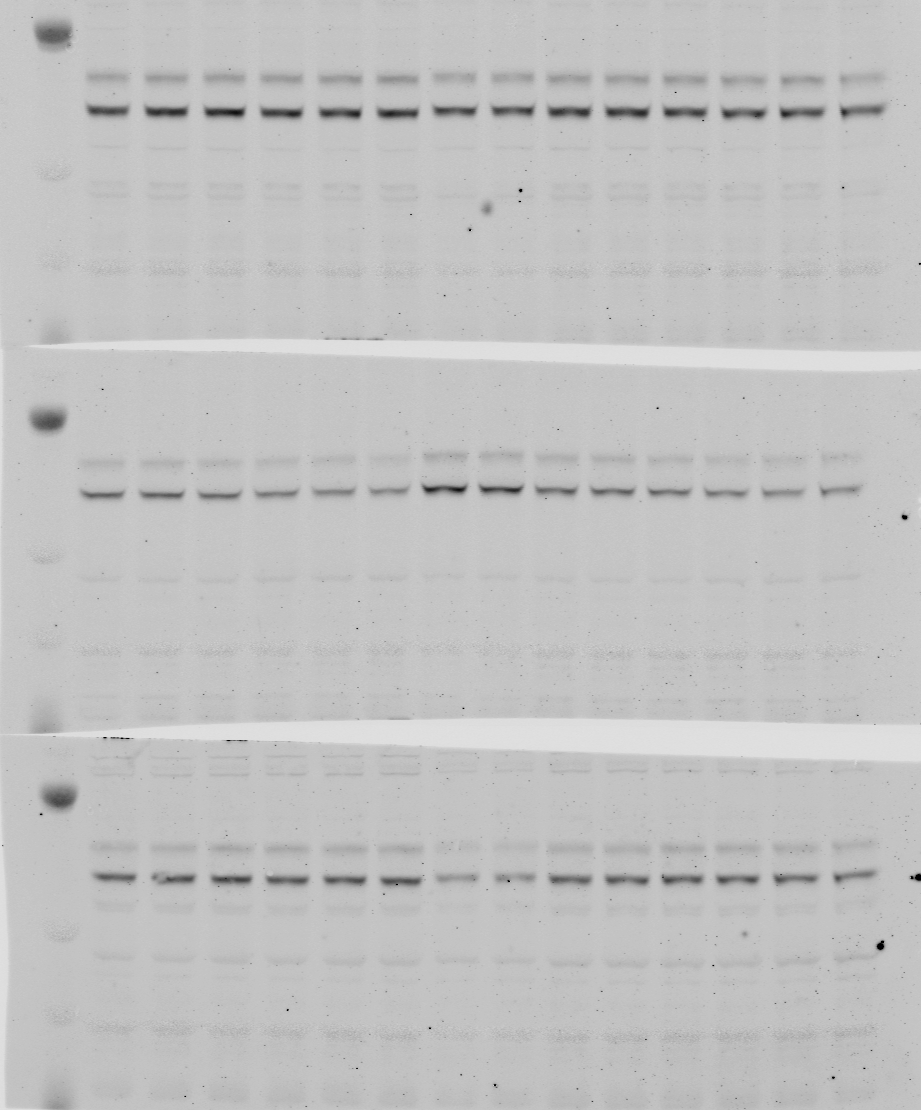

Supplement: Figure 3—source data 1. [file elife-103403-fig3-data1.zip › Figures_3D_and_S4C-western_blots/16Feb2024 S6K p371-389-421.png]

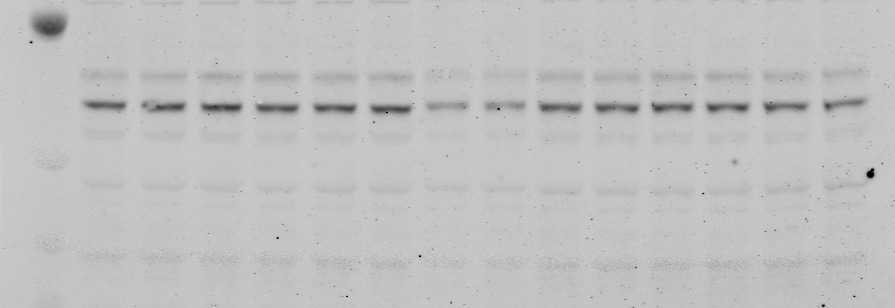

Supplement: Figure 3—source data 1. [file elife-103403-fig3-data1.zip › Figures_3D_and_S4C-western_blots/16Feb2024 S6K p371.png]

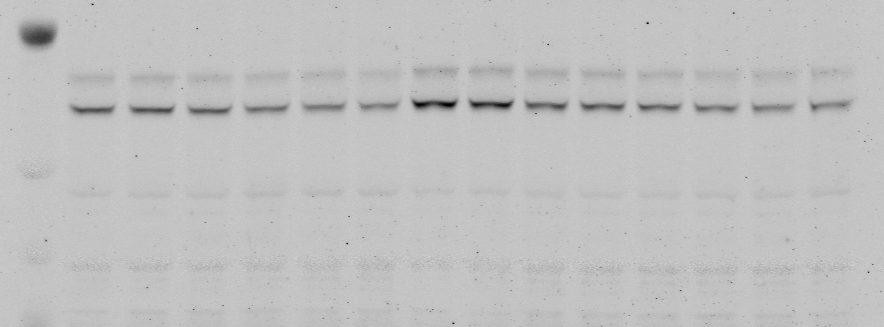

Supplement: Figure 3—source data 1. [file elife-103403-fig3-data1.zip › Figures_3D_and_S4C-western_blots/16Feb2024 S6K p389.png]

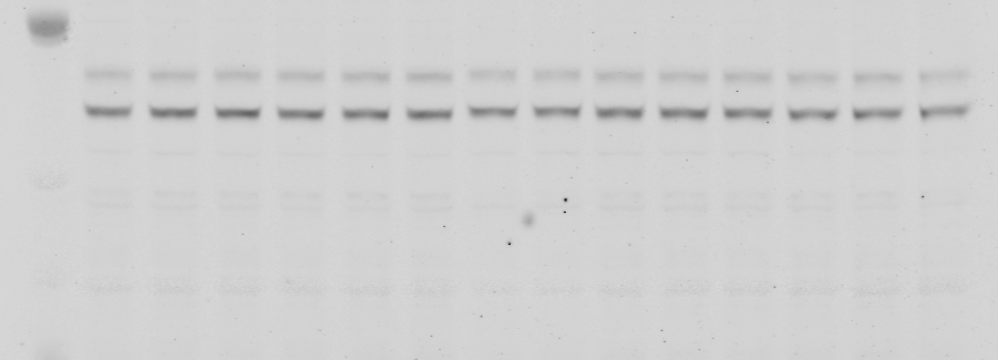

Supplement: Figure 3—source data 1. [file elife-103403-fig3-data1.zip › Figures_3D_and_S4C-western_blots/16Feb2024 S6K p421.png]

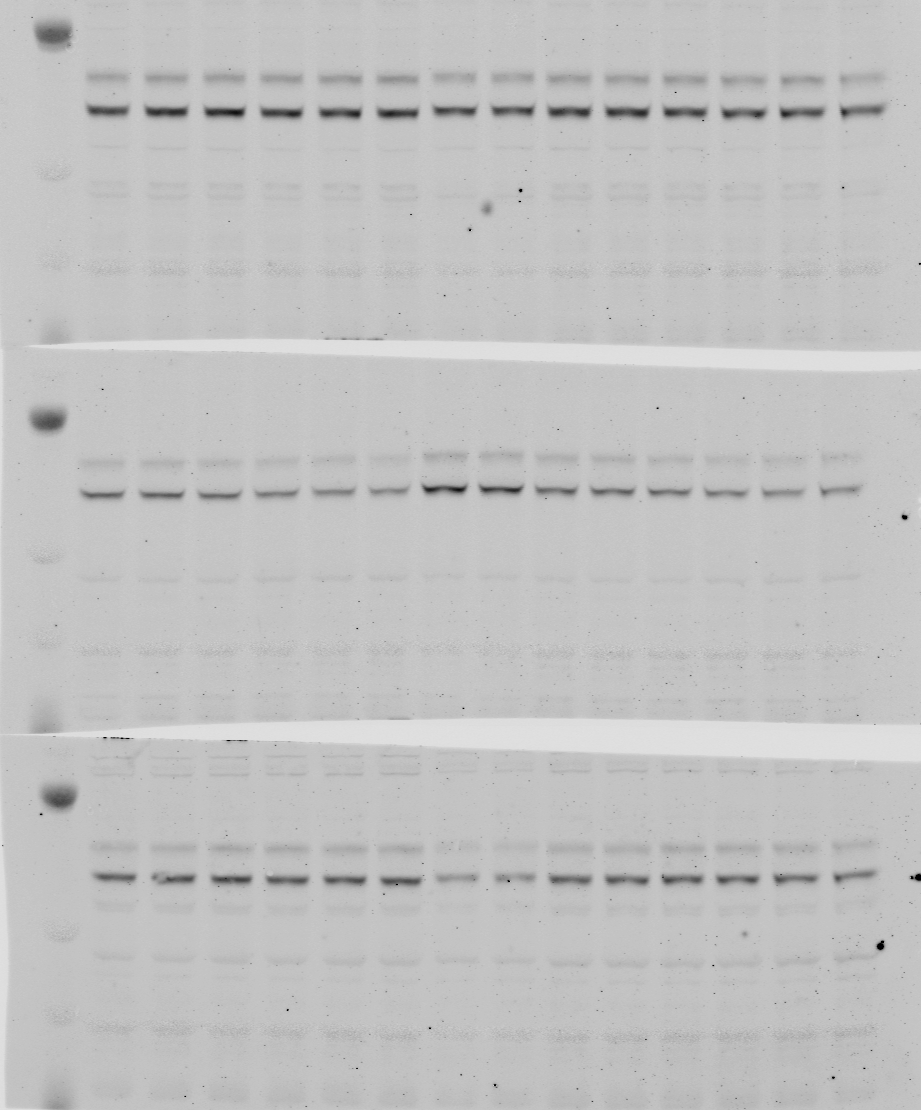

Supplement: Figure 3—source data 1. [file elife-103403-fig3-data1.zip › Figures_3D_and_S4C-western_blots/16Feb2024 S6K total.tif]

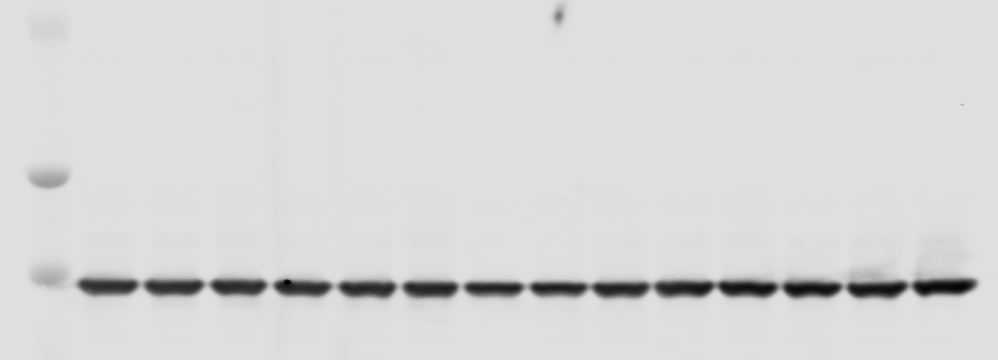

Supplement: Figure 3—source data 1. [file elife-103403-fig3-data1.zip › Figures_3D_and_S4C-western_blots/16Feb2024 S6K-GAPDH.png]

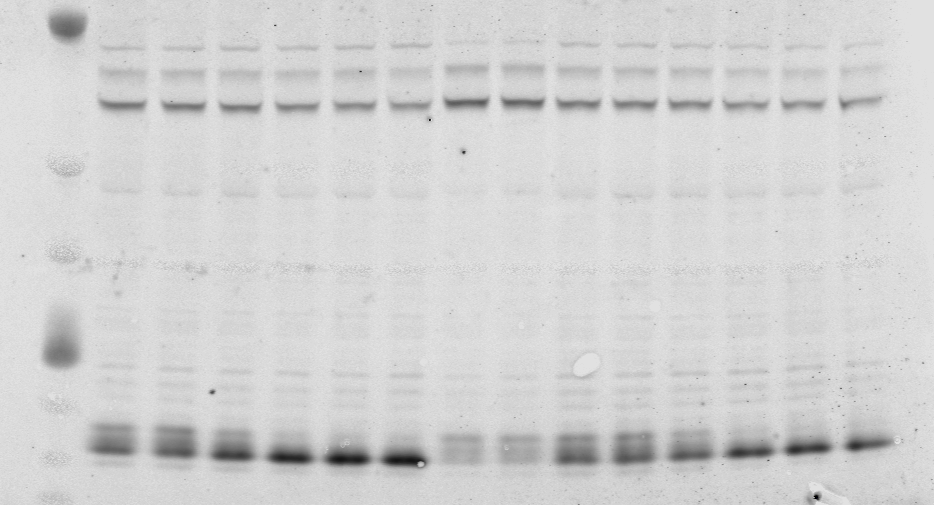

Supplement: Figure 3—source data 1. [file elife-103403-fig3-data1.zip › Figures_3D_and_S4C-western_blots/16Feb2024 t4EBP.png]

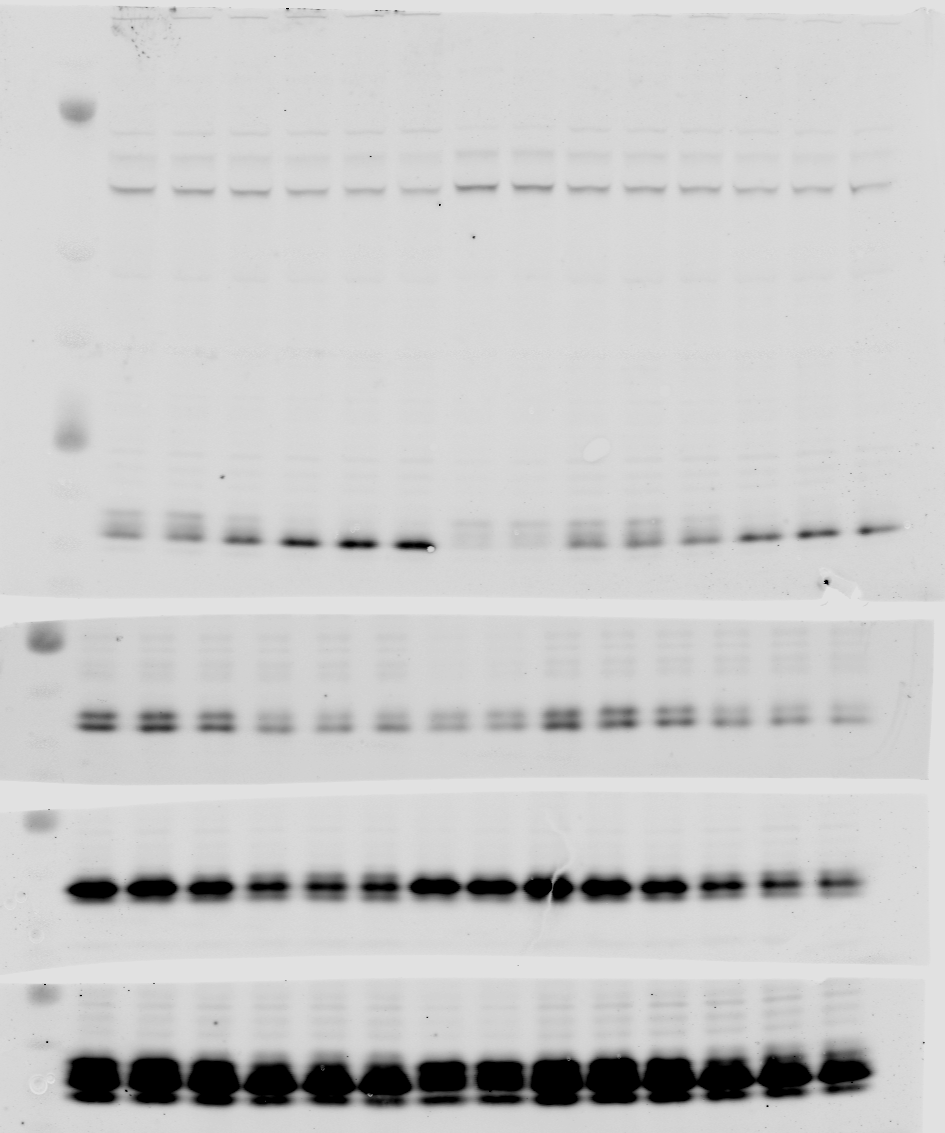

Supplement: Figure 3—source data 1. [file elife-103403-fig3-data1.zip › Figures_3D_and_S4C-western_blots/16Feb2024 t4EBP1, 4EBP1 p70-65-37.png]

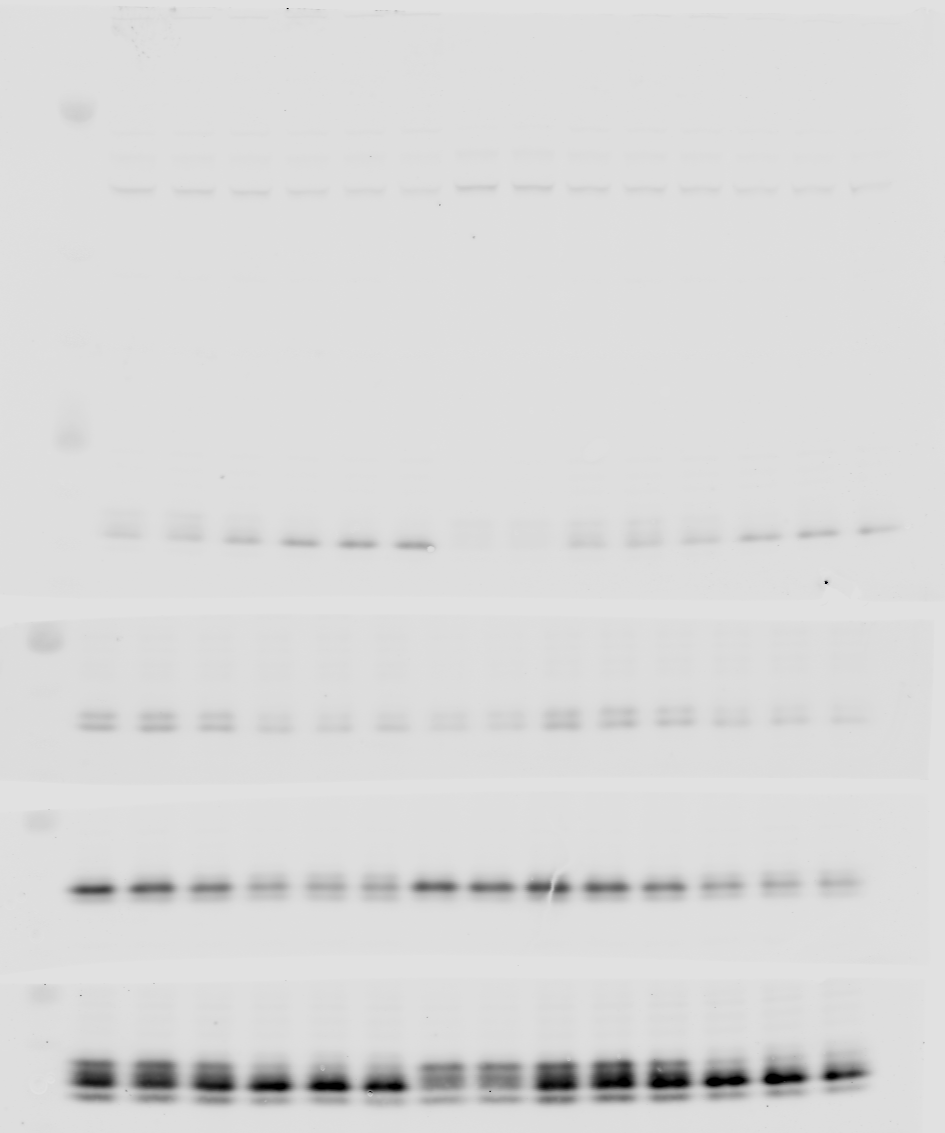

Supplement: Figure 3—source data 1. [file elife-103403-fig3-data1.zip › Figures_3D_and_S4C-western_blots/16Feb2024 t4EBP1, 4EBP1 p70-65-37.tif]

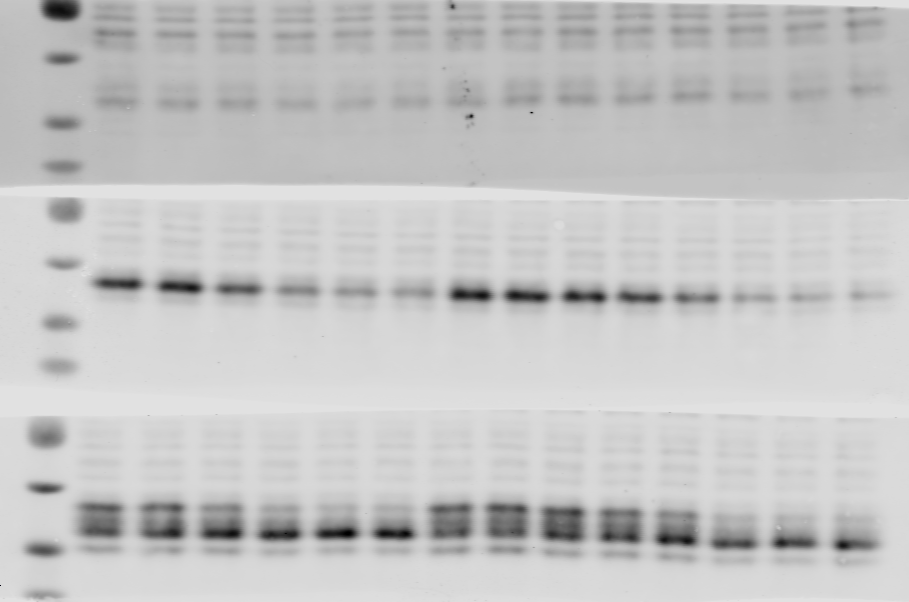

Supplement: Figure 3—source data 1. [file elife-103403-fig3-data1.zip › Figures_3D_and_S4C-western_blots/22Mar2024 4EBP1 p37-65-70.png]

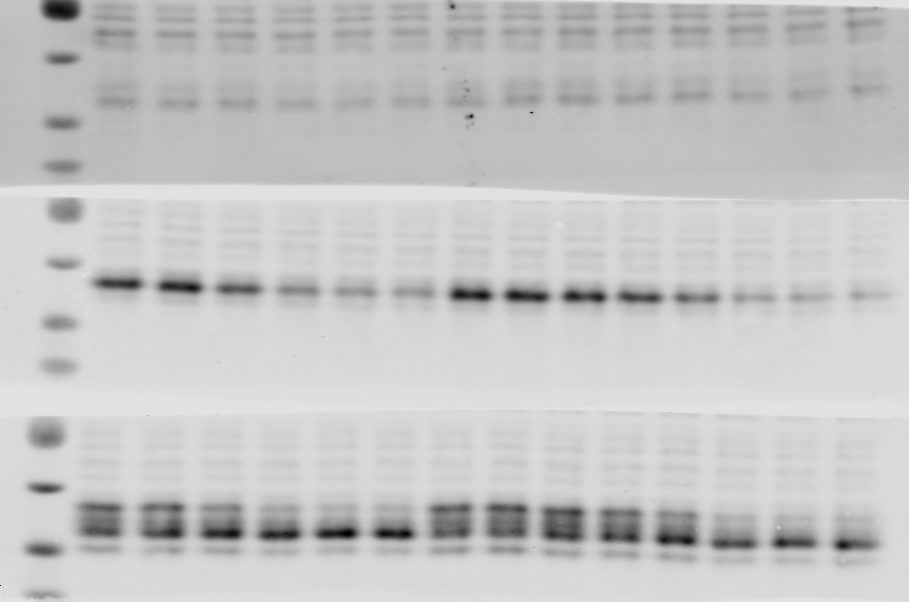

Supplement: Figure 3—source data 1. [file elife-103403-fig3-data1.zip › Figures_3D_and_S4C-western_blots/22Mar2024 4EBP1 p37-65-70.tif]

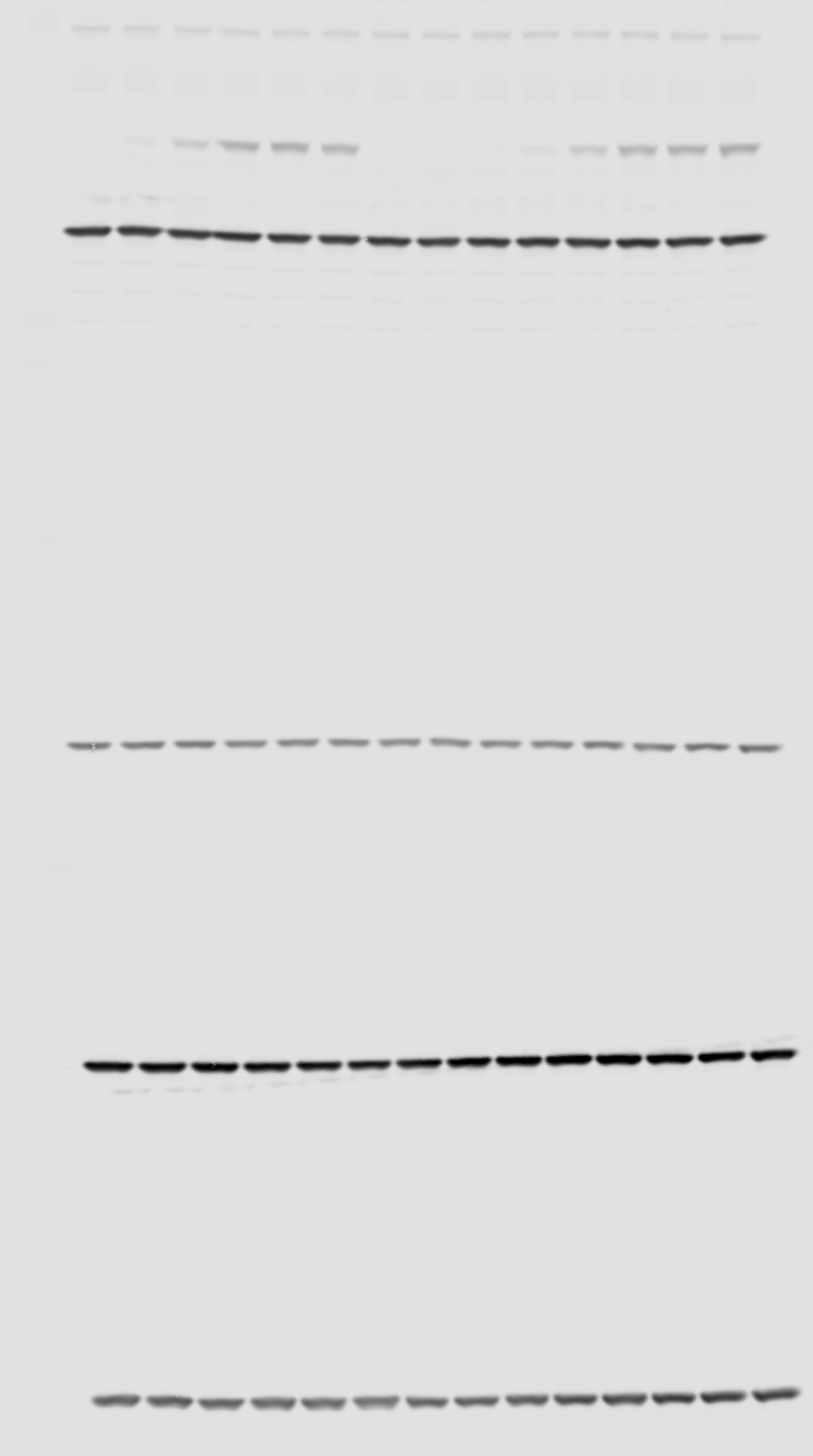

Supplement: Figure 3—source data 1. [file elife-103403-fig3-data1.zip › Figures_3D_and_S4C-western_blots/22Mar2024 GAPDH-FLAG-389M.tif]

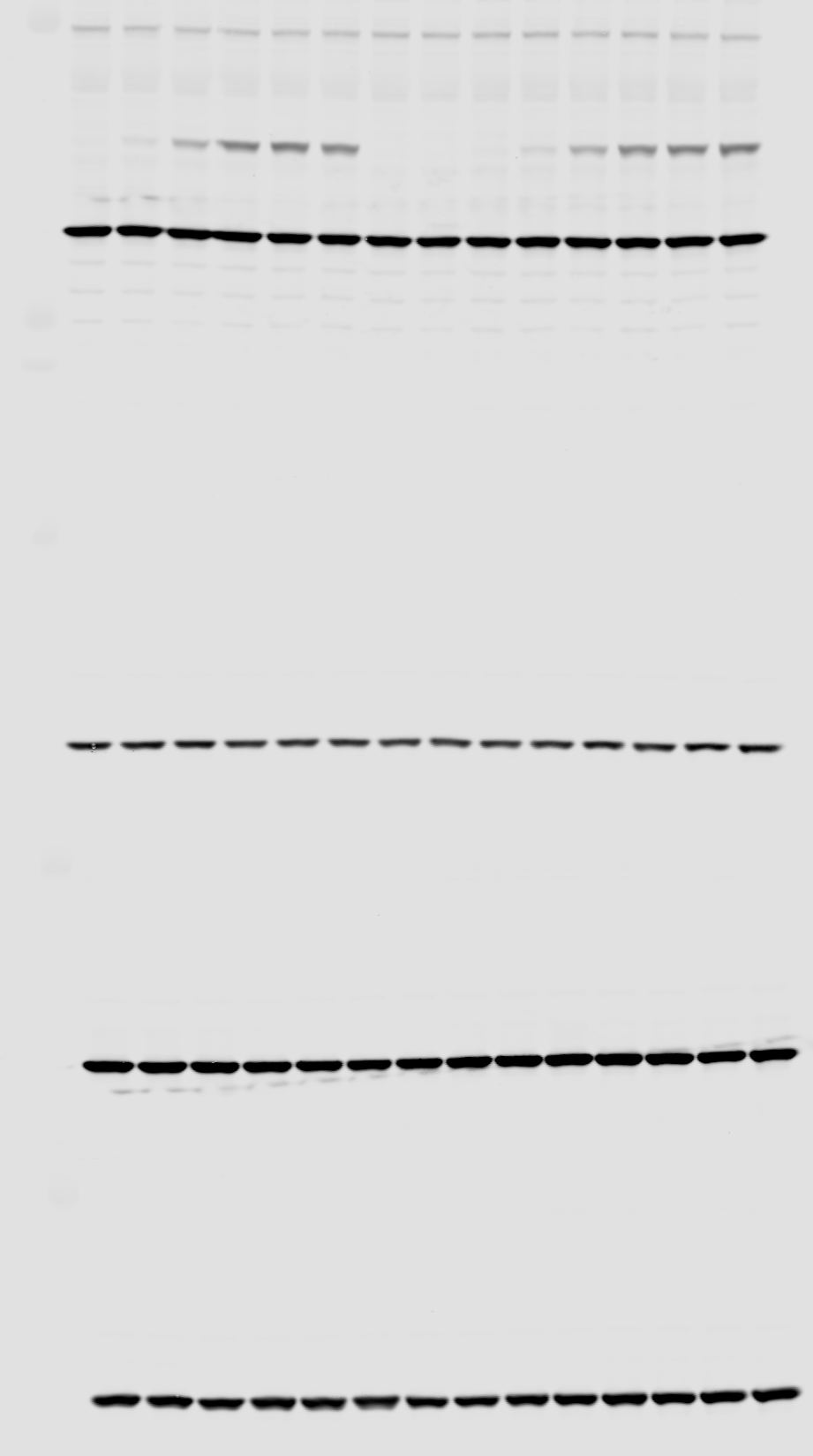

Supplement: Figure 3—source data 1. [file elife-103403-fig3-data1.zip › Figures_3D_and_S4C-western_blots/22Mar2024 GAPDH-FLAG.png]

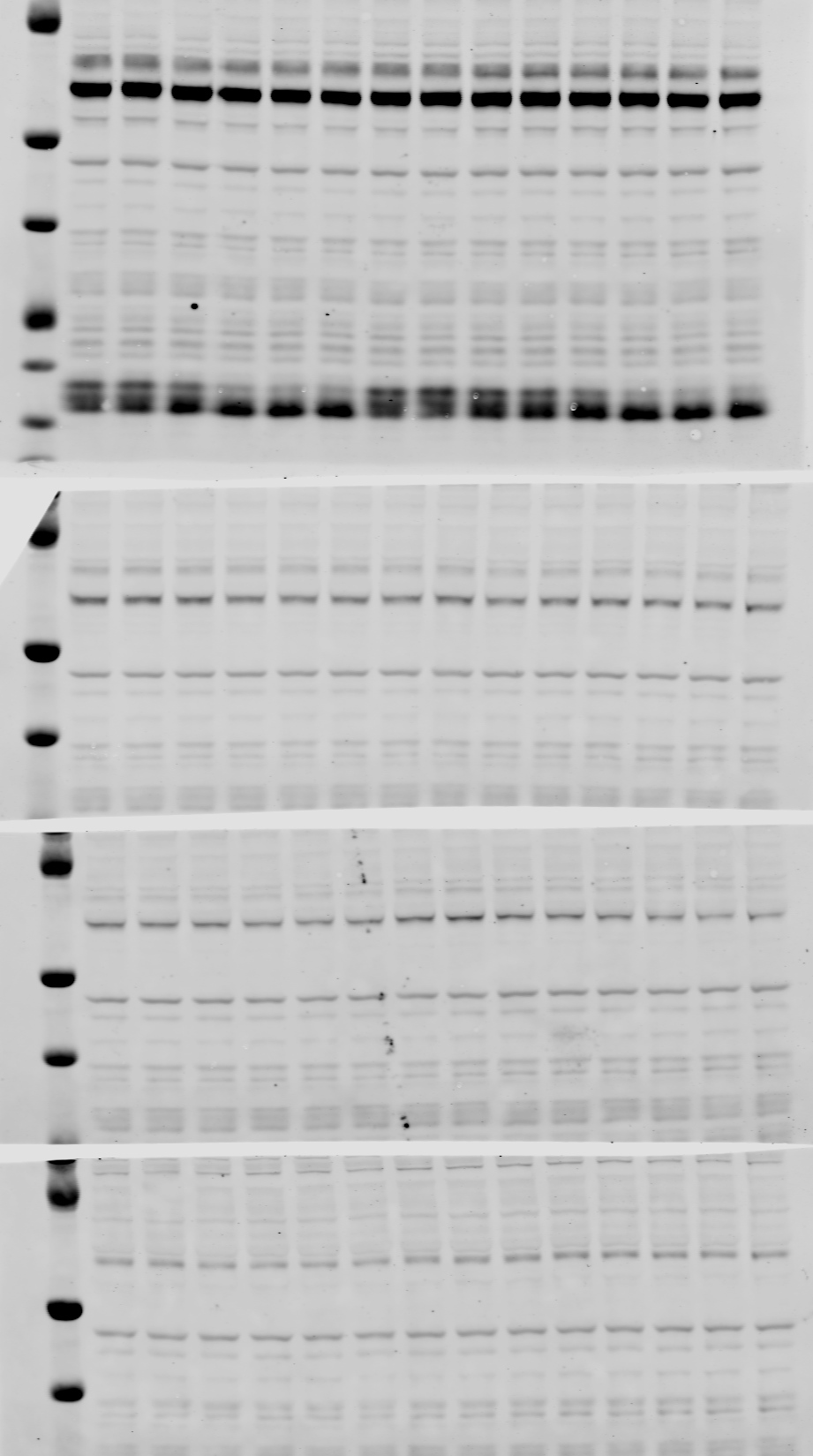

Supplement: Figure 3—source data 1. [file elife-103403-fig3-data1.zip › Figures_3D_and_S4C-western_blots/22Mar2024 S6K p371-389-421, 4ebpT, S6KT.png]

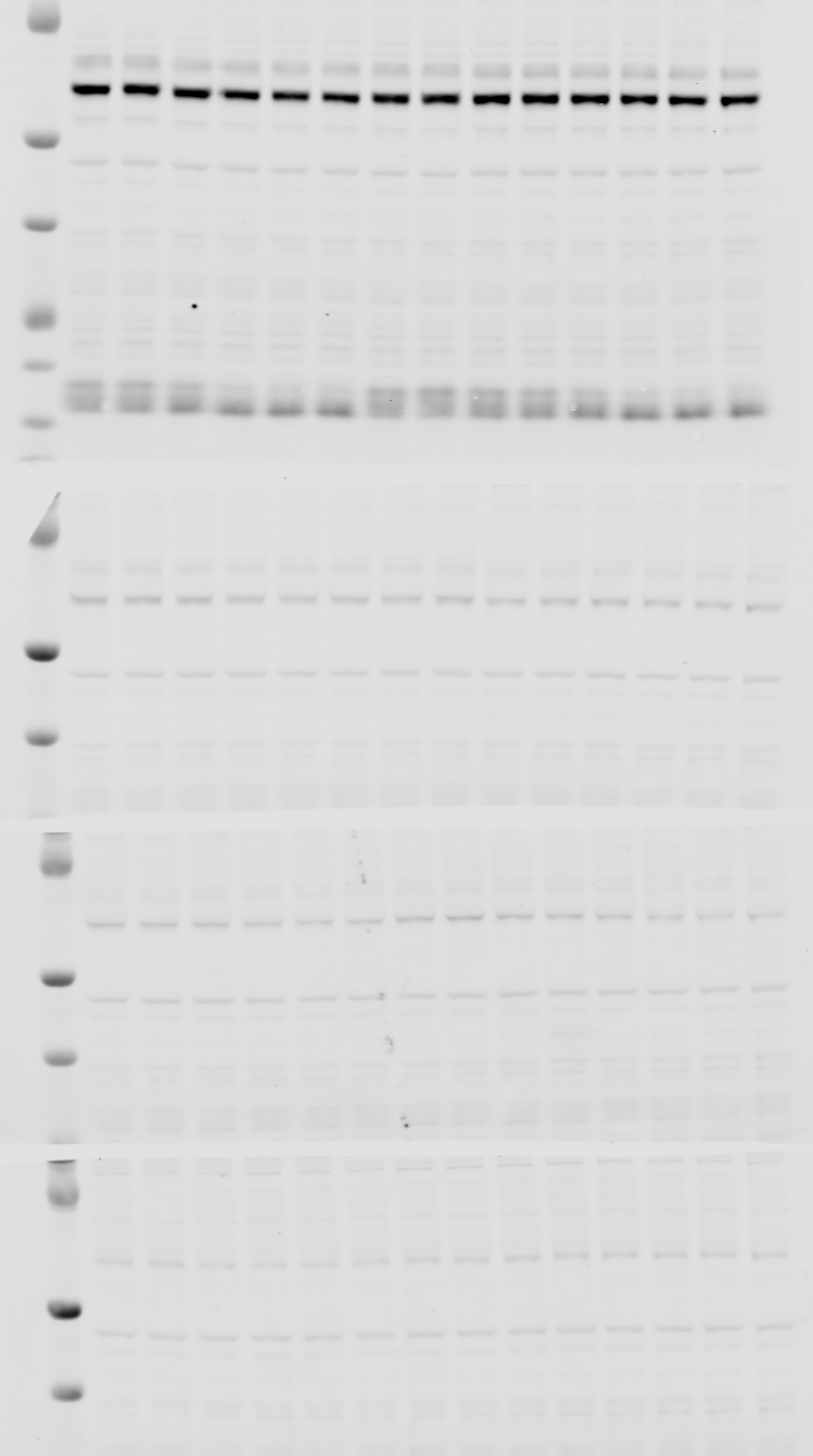

Supplement: Figure 3—source data 1. [file elife-103403-fig3-data1.zip › Figures_3D_and_S4C-western_blots/22Mar2024 S6K p371-389-421, 4ebpT, S6KT.tif]

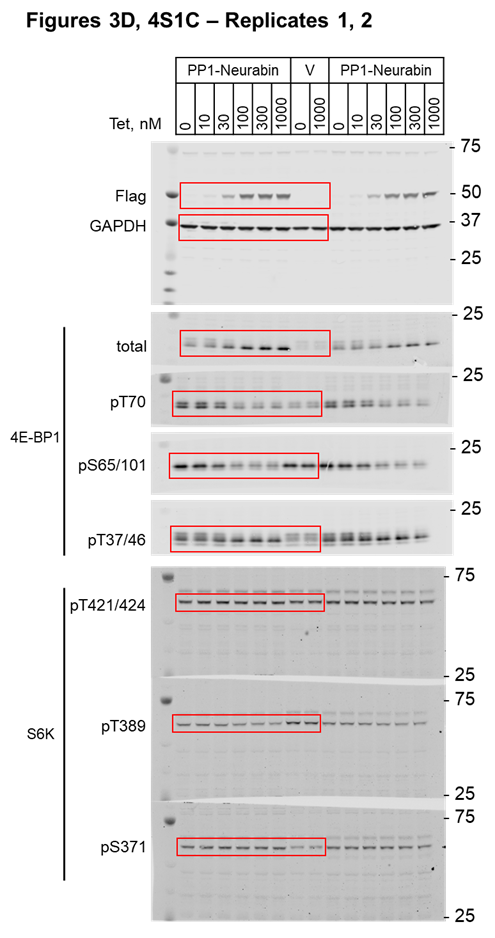

Supplement: Figure 3—source data 1. [file elife-103403-fig3-data1.zip › Figure 3D-1.png]

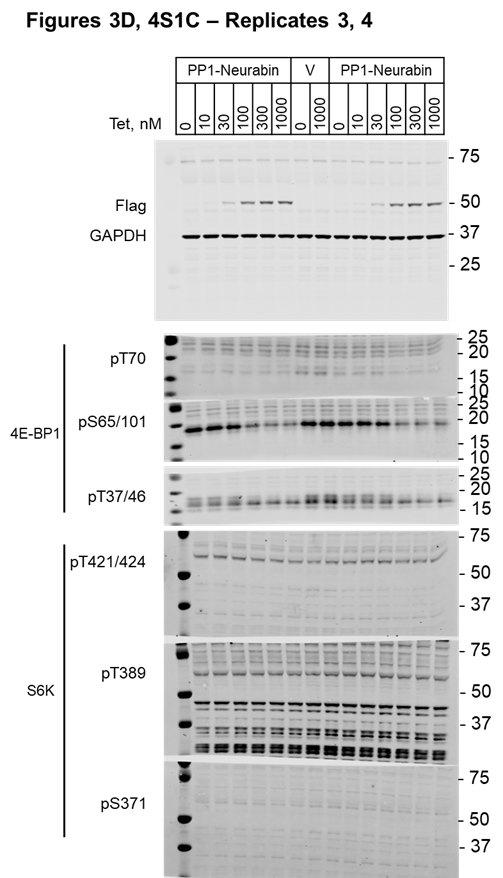

Supplement: Figure 3—source data 1. [file elife-103403-fig3-data1.zip › Figure 3D-2.png]

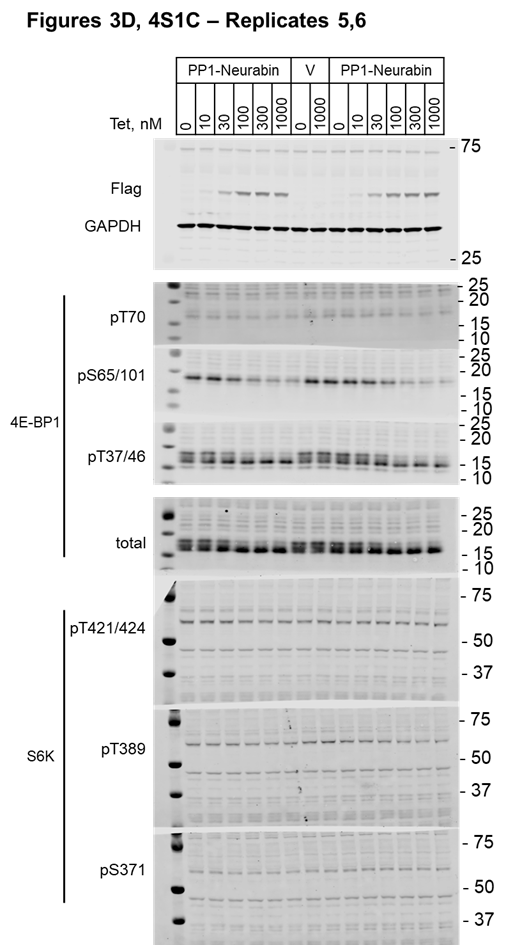

Supplement: Figure 3—source data 1. [file elife-103403-fig3-data1.zip › Figure 3D-3.png]

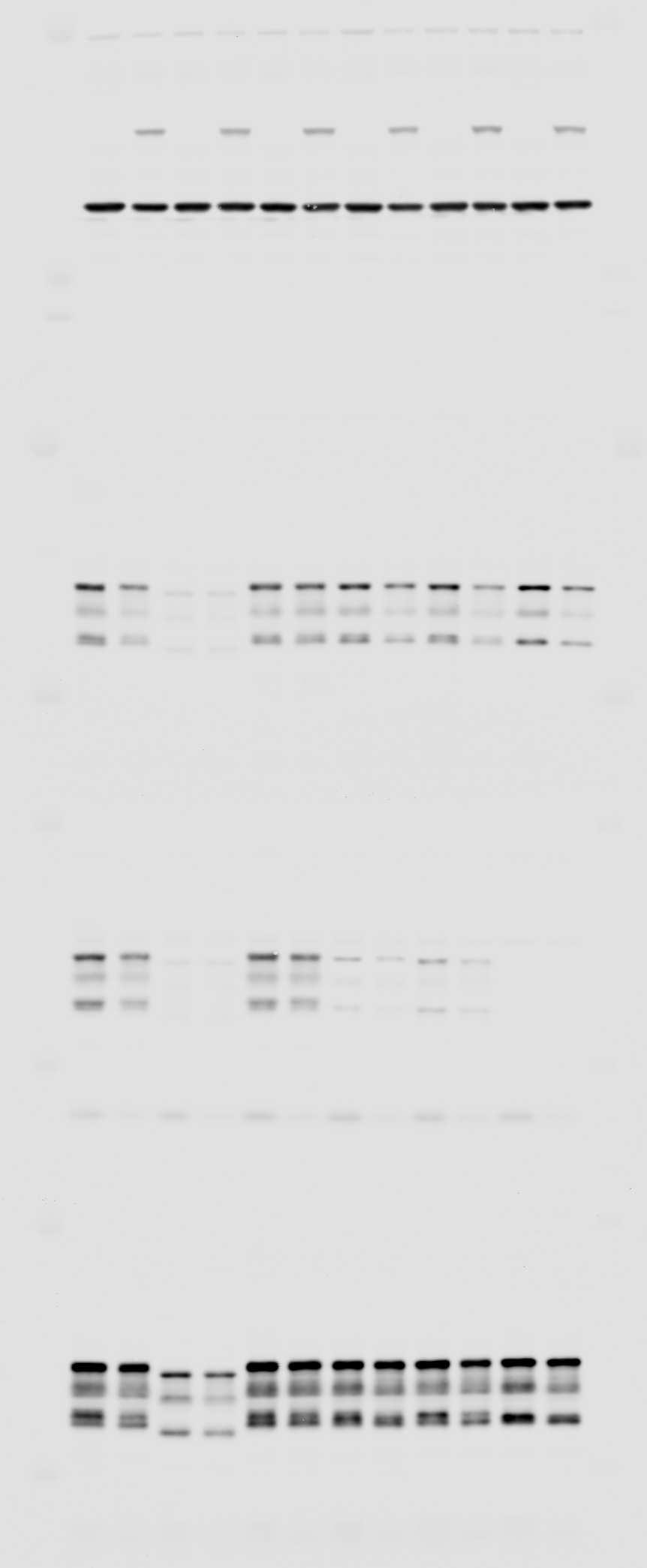

Supplement: Figure 3—figure supplement 1—source data 1. [file elife-103403-fig3-figsupp1-data1.zip › Figures_S3F_S4A-western_blots/13Mar2024 37-65-70--GAPDH-FLAG.png]

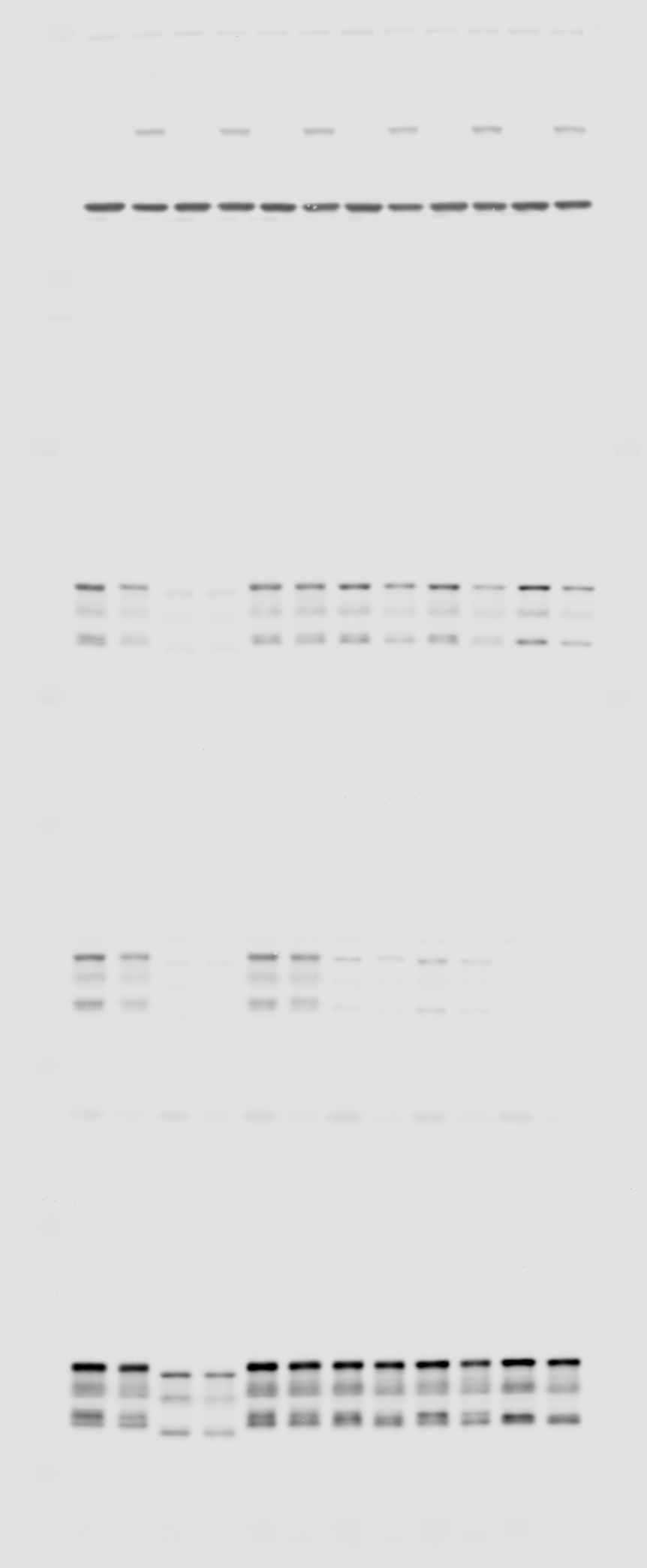

Supplement: Figure 3—figure supplement 1—source data 1. [file elife-103403-fig3-figsupp1-data1.zip › Figures_S3F_S4A-western_blots/13Mar2024 37-65-70--GAPDH-FLAG.tif]

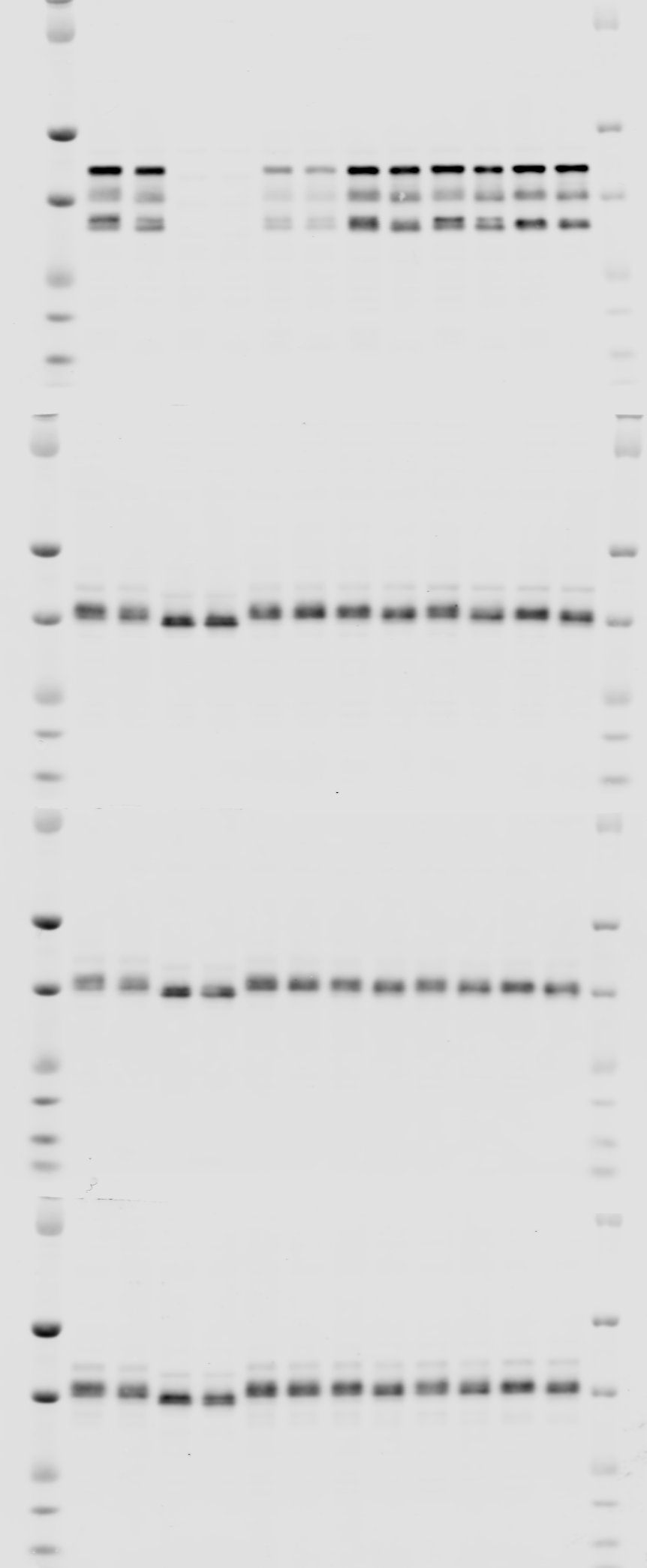

Supplement: Figure 3—figure supplement 1—source data 1. [file elife-103403-fig3-figsupp1-data1.zip › Figures_S3F_S4A-western_blots/13Mar2024 mCherryx3-4EBPT.tif]

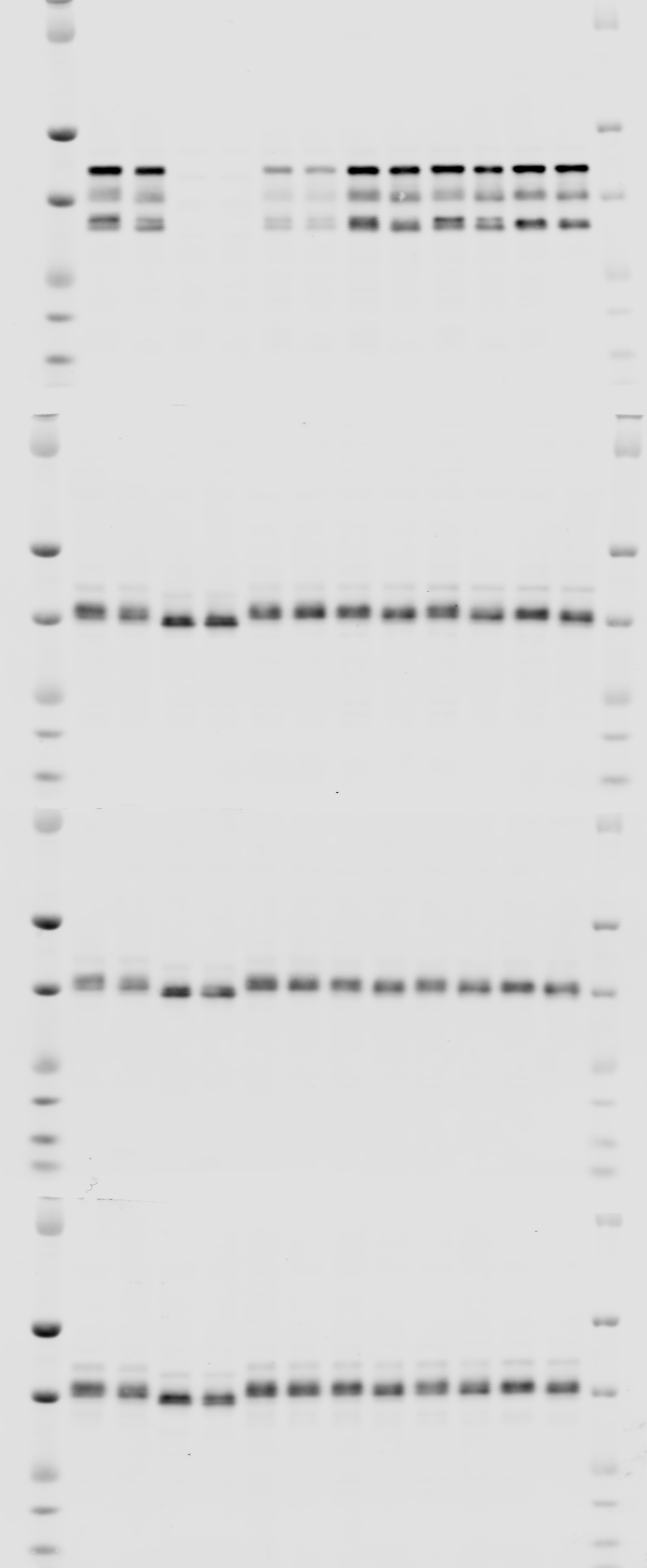

Supplement: Figure 3—figure supplement 1—source data 1. [file elife-103403-fig3-figsupp1-data1.zip › Figures_S3F_S4A-western_blots/13Mar2024 mCHerryx3-4EBPTOT.png]

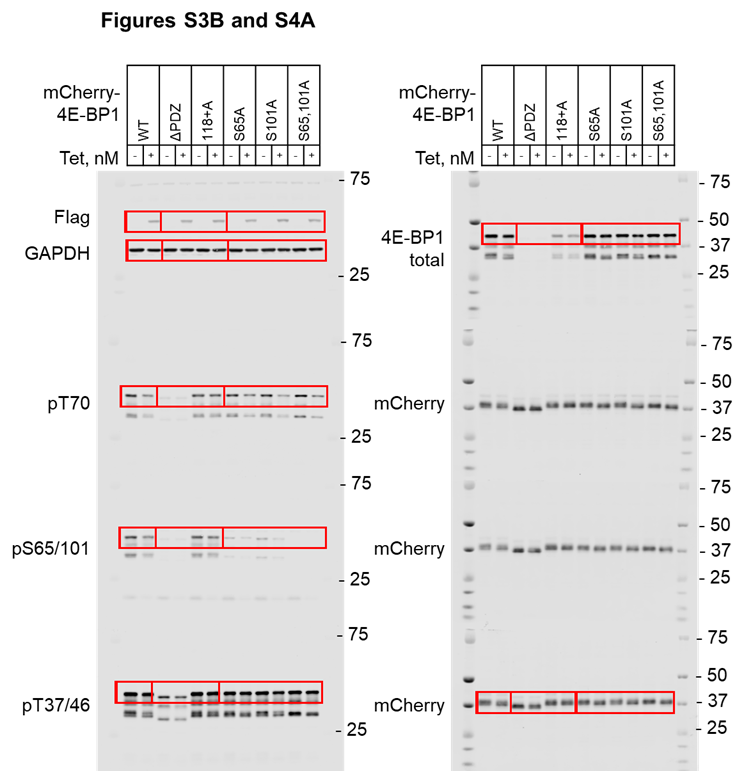

Supplement: Figure 3—figure supplement 1—source data 1. [file elife-103403-fig3-figsupp1-data1.zip › Figure S3B S4A.png]

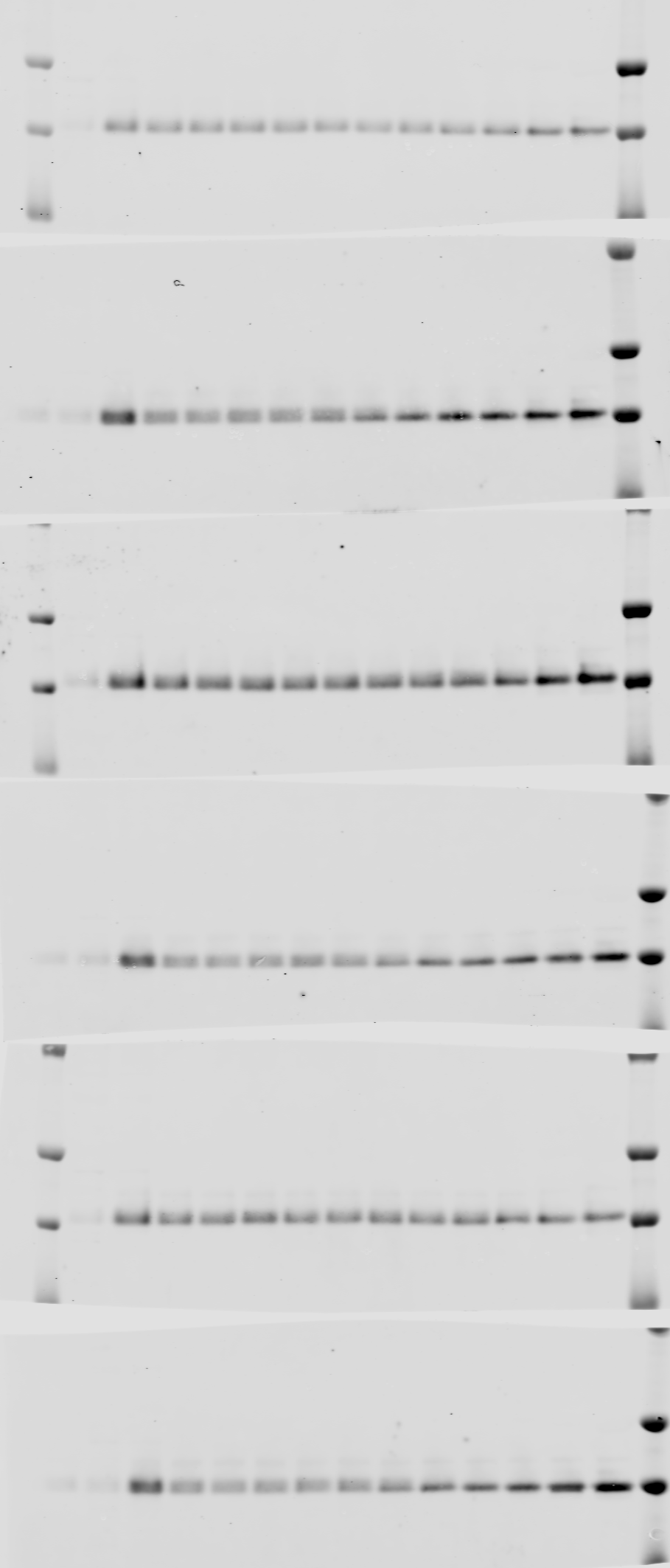

Supplement: Figure 4—source data 1. [file elife-103403-fig4-data1.zip › Figure_4A,4B-western_blots/8Dec2024_4EBP1-mCherry.png]

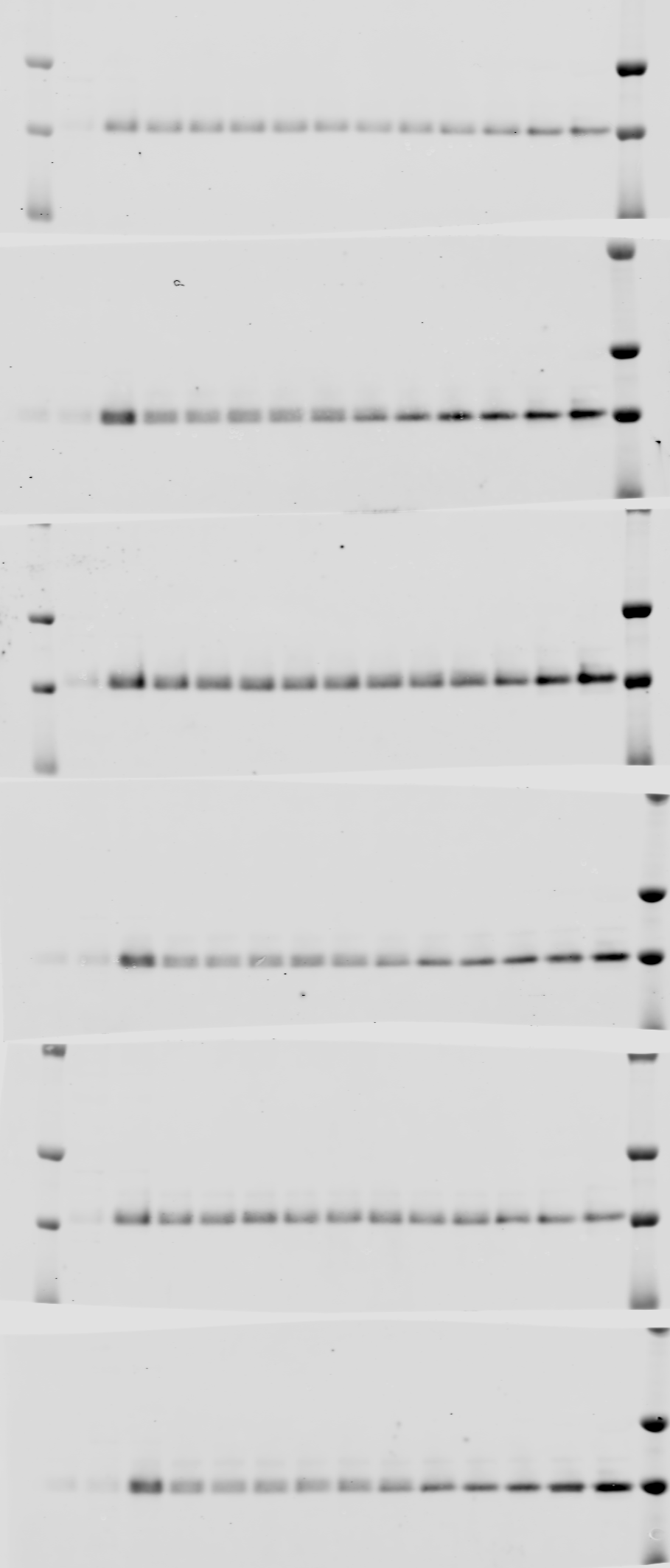

Supplement: Figure 4—source data 1. [file elife-103403-fig4-data1.zip › Figure_4A,4B-western_blots/8Dec2024_4EBP1-mCherry.tif]

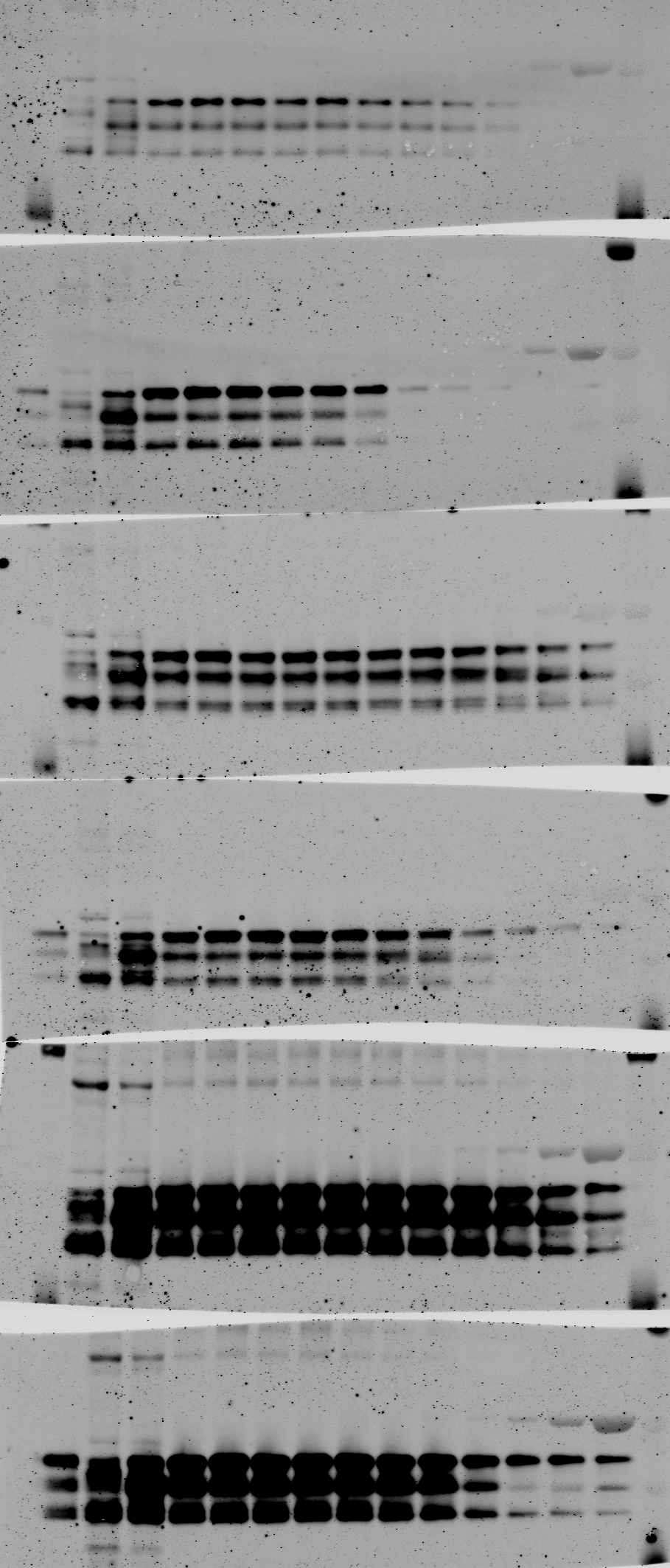

Supplement: Figure 4—source data 1. [file elife-103403-fig4-data1.zip › Figure_4A,4B-western_blots/8Dec2024_4EBP1-WT-and-118A-p70-65-37-high_contrast.png]

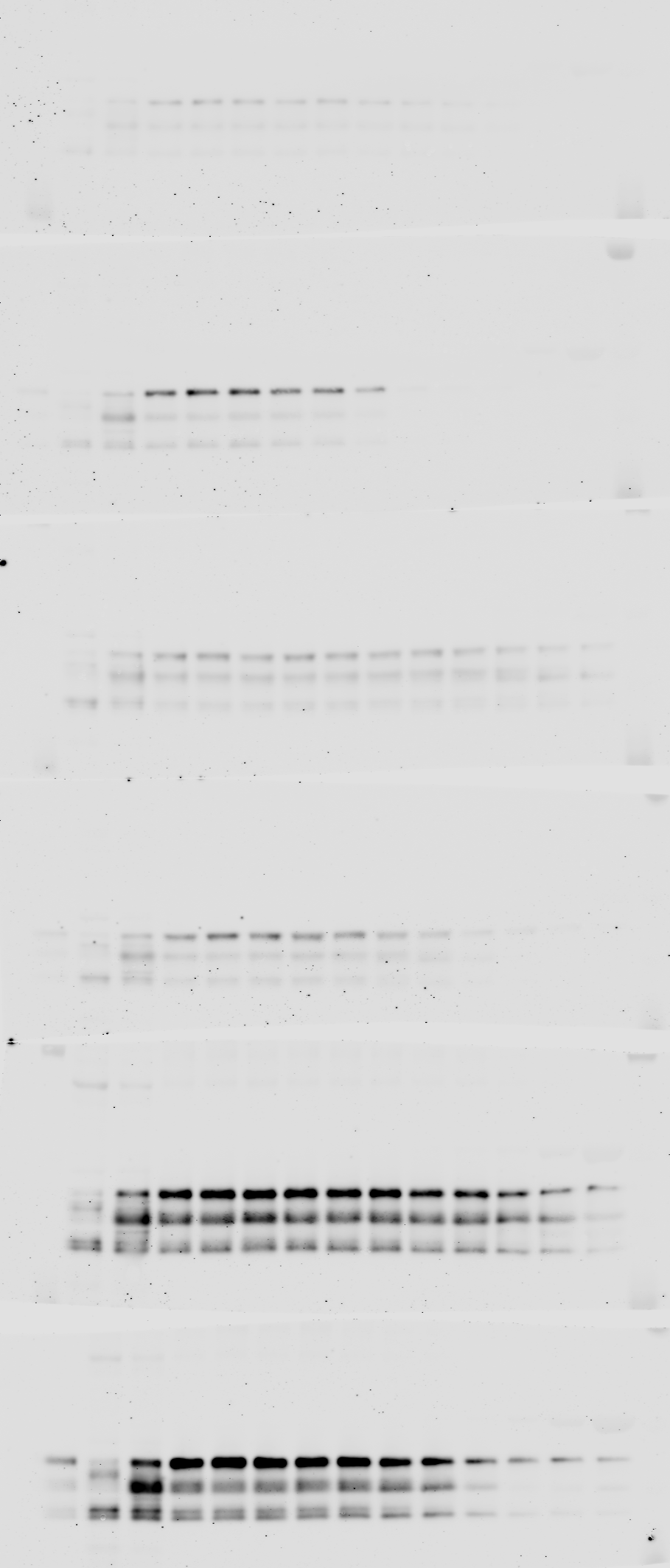

Supplement: Figure 4—source data 1. [file elife-103403-fig4-data1.zip › Figure_4A,4B-western_blots/8Dec2024_4EBP1-WT-and-118A-p70-65-37-low_contrast.png]

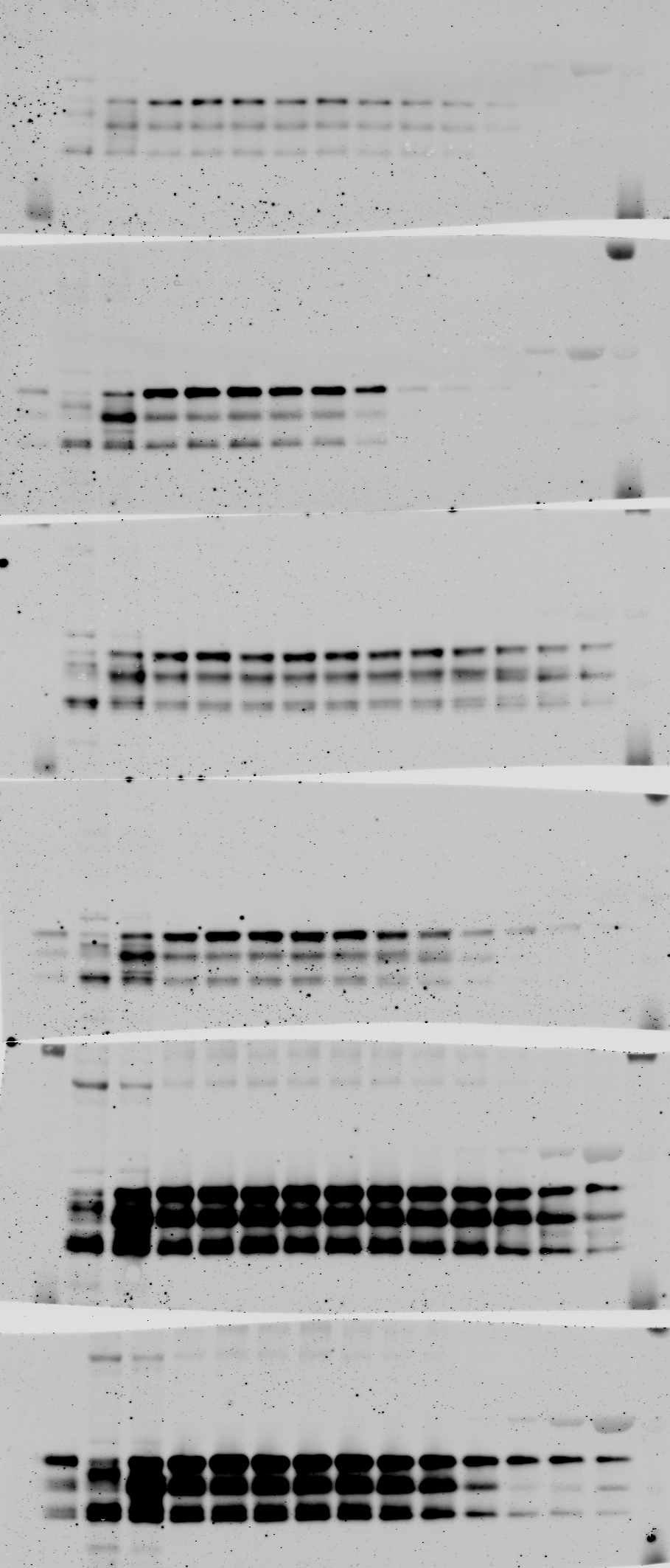

Supplement: Figure 4—source data 1. [file elife-103403-fig4-data1.zip › Figure_4A,4B-western_blots/8Dec2024_4EBP1-WT-and-118A-p70-65-37-medium_contrast.png]

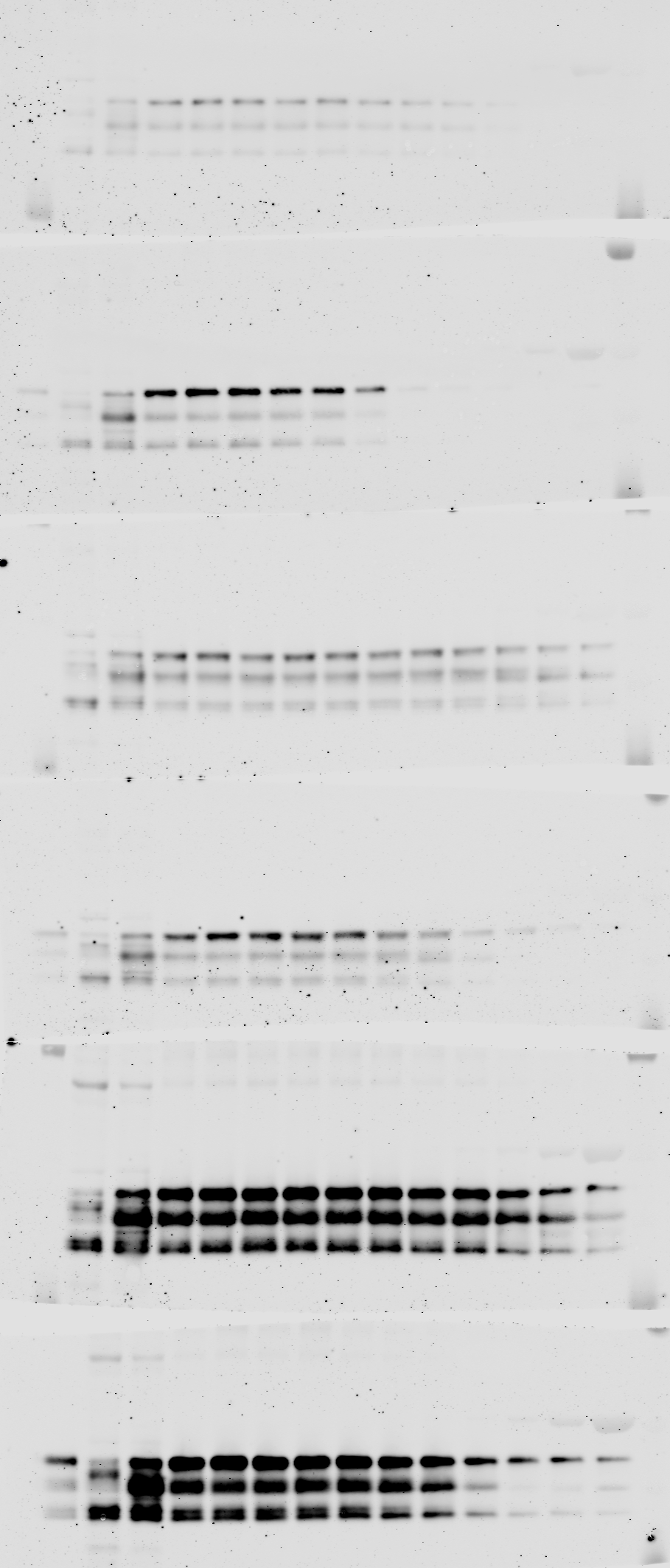

Supplement: Figure 4—source data 1. [file elife-103403-fig4-data1.zip › Figure_4A,4B-western_blots/8Dec2024_4EBP1-WT-and-118A-p70-65-37-original.tif]

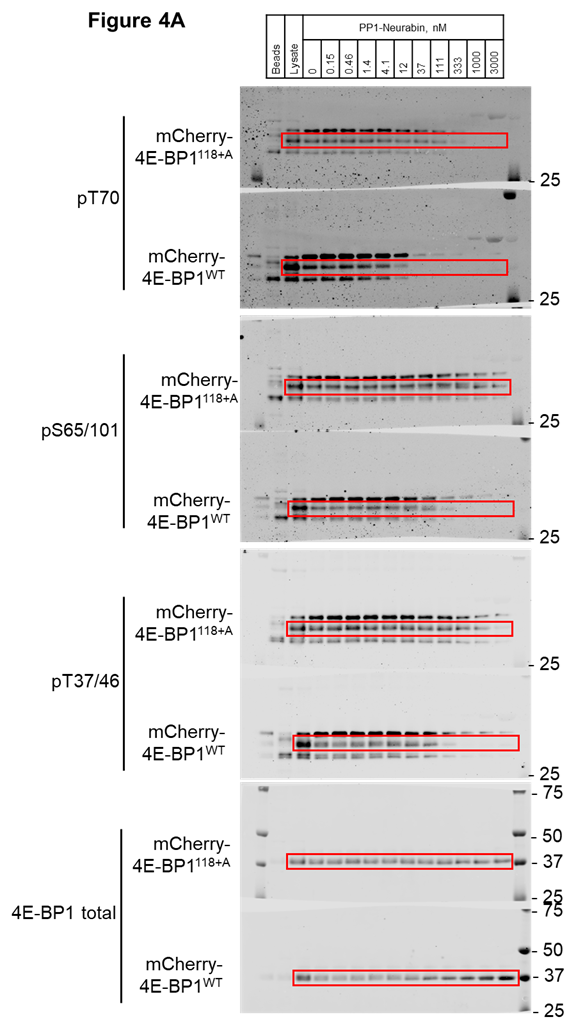

Supplement: Figure 4—source data 1. [file elife-103403-fig4-data1.zip › Figure 4A.png]
