# Supplementary material for: PDZ-directed substrate recruitment is the primary determinant of specific 4E-BP1 dephosphorylation by PP1-Neurabin
Source: eLife. 2025 Jun 23;13:RP103403. doi: 10.7554/eLife.103403 (PMC12185105; doi:10.7554/eLife.103403)
Supplement: Figure 1—source data 5. [file elife-103403-fig1-data5.zip › Western Blots 1C.pptx]

## Slide 1
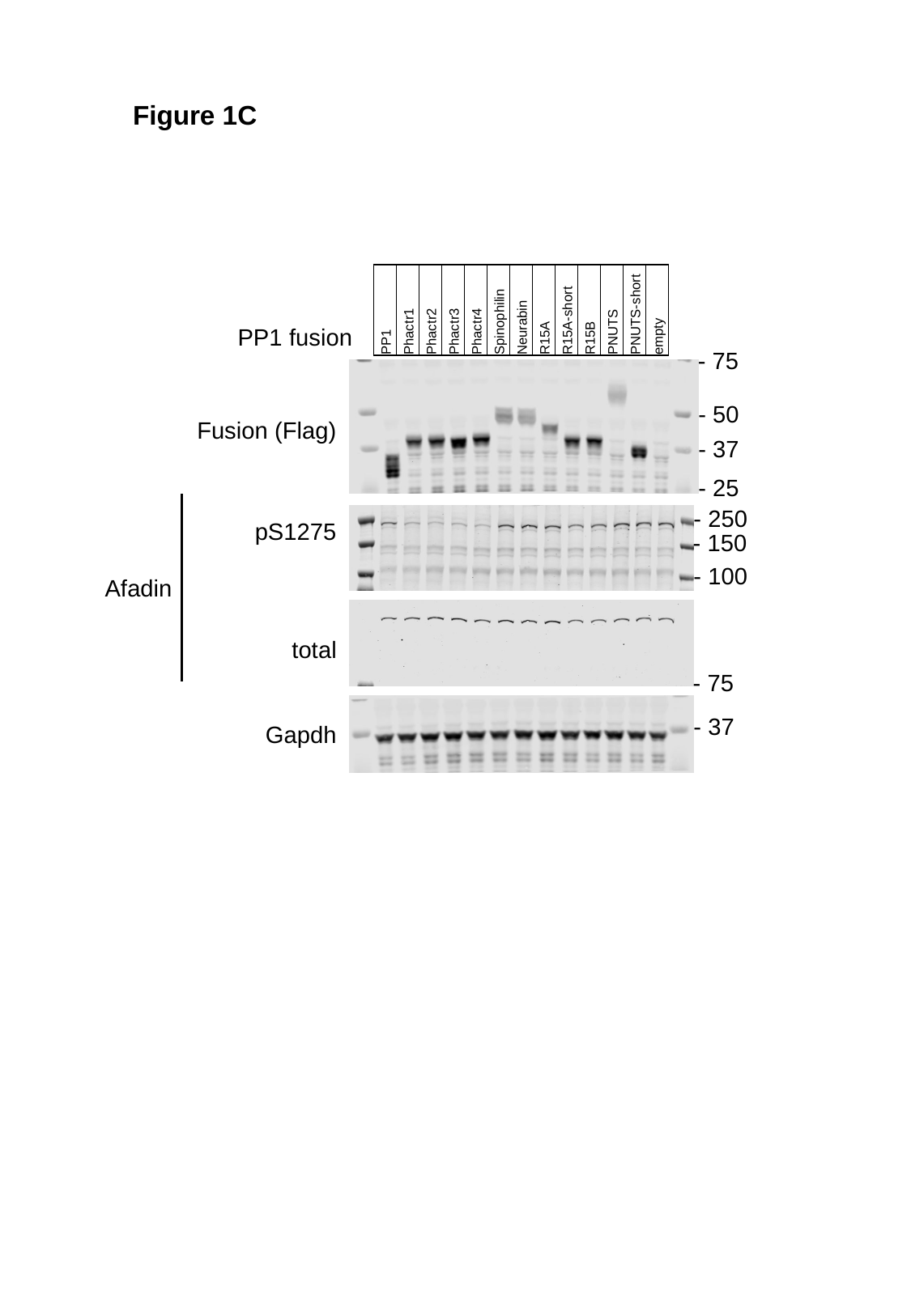

Figure 1C
| PP1 | Phactr1 | Phactr2 | Phactr3 | Phactr4 | Spinophilin | Neurabin | R15A | R15A-short | R15B | PNUTS | PNUTS-short | empty |
| --- | --- | --- | --- | --- | --- | --- | --- | --- | --- | --- | --- | --- |
PP1 fusion
- 75
- 50
Fusion (Flag)
- 37
- 25
- 250
pS1275
- 150
- 100
Afadin
total
- 75
- 37
Gapdh
