# Supplementary material for: PDZ-directed substrate recruitment is the primary determinant of specific 4E-BP1 dephosphorylation by PP1-Neurabin
Source: eLife. 2025 Jun 23;13:RP103403. doi: 10.7554/eLife.103403 (PMC12185105; doi:10.7554/eLife.103403)
Supplement: Figure 3—source data 1. [file elife-103403-fig3-data1.zip › Western Blots 3D,4-S1C.pptx]

## Slide 1
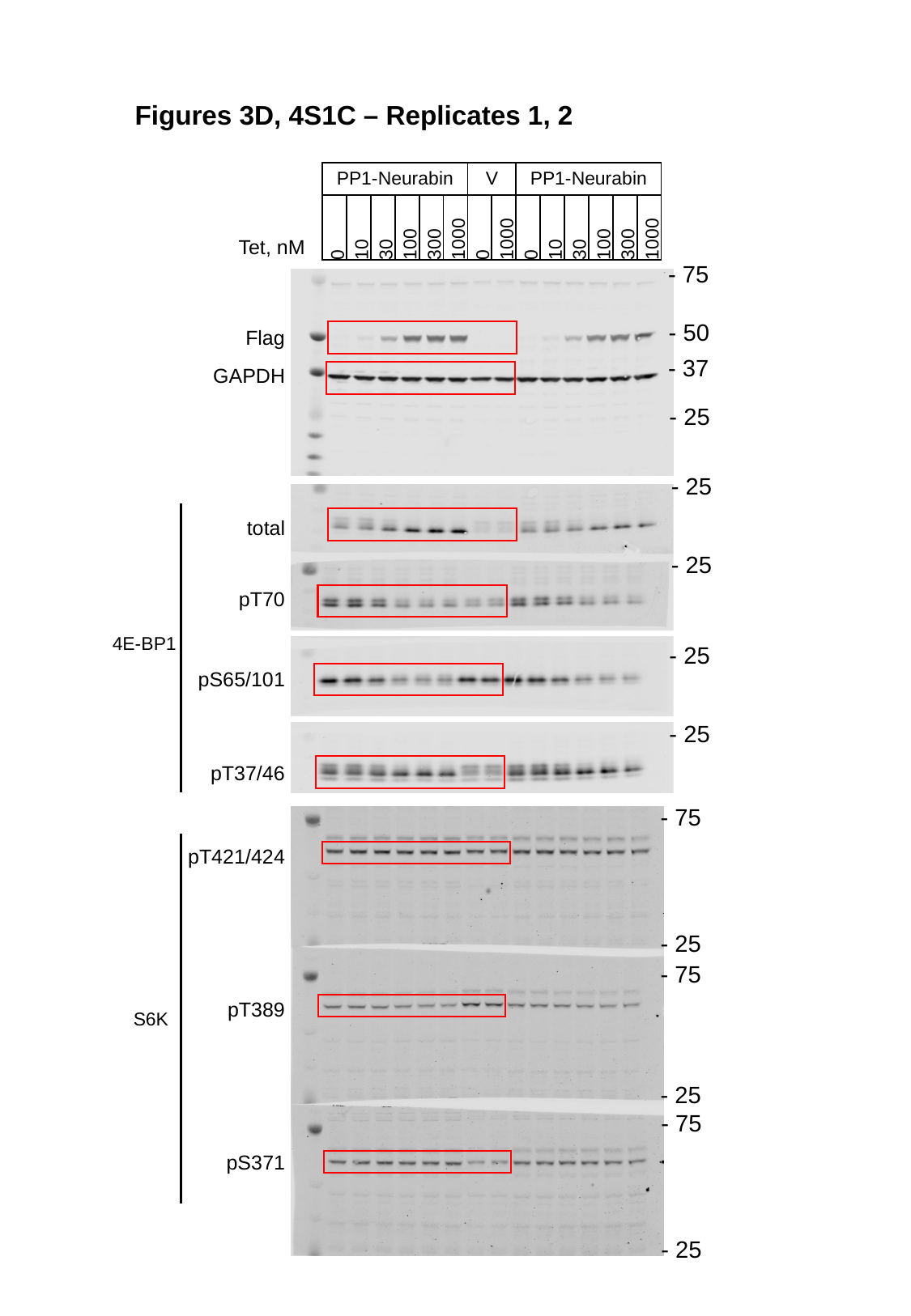

Figures 3D, 4S1C – Replicates 1, 2
| PP1-Neurabin | | | | | | V | | PP1-Neurabin | | | | | |
| --- | --- | --- | --- | --- | --- | --- | --- | --- | --- | --- | --- | --- | --- |
| 0 | 10 | 30 | 100 | 300 | 1000 | 0 | 1000 | 0 | 10 | 30 | 100 | 300 | 1000 |
Tet, nM
- 75
- 50
Flag
- 37
GAPDH
- 25
- 25
total
- 25
pT70
4E-BP1
- 25
pS65/101
- 25
pT37/46
- 75
pT421/424
- 25
- 75
pT389
S6K
- 25
- 75
pS371
- 25

## Slide 2
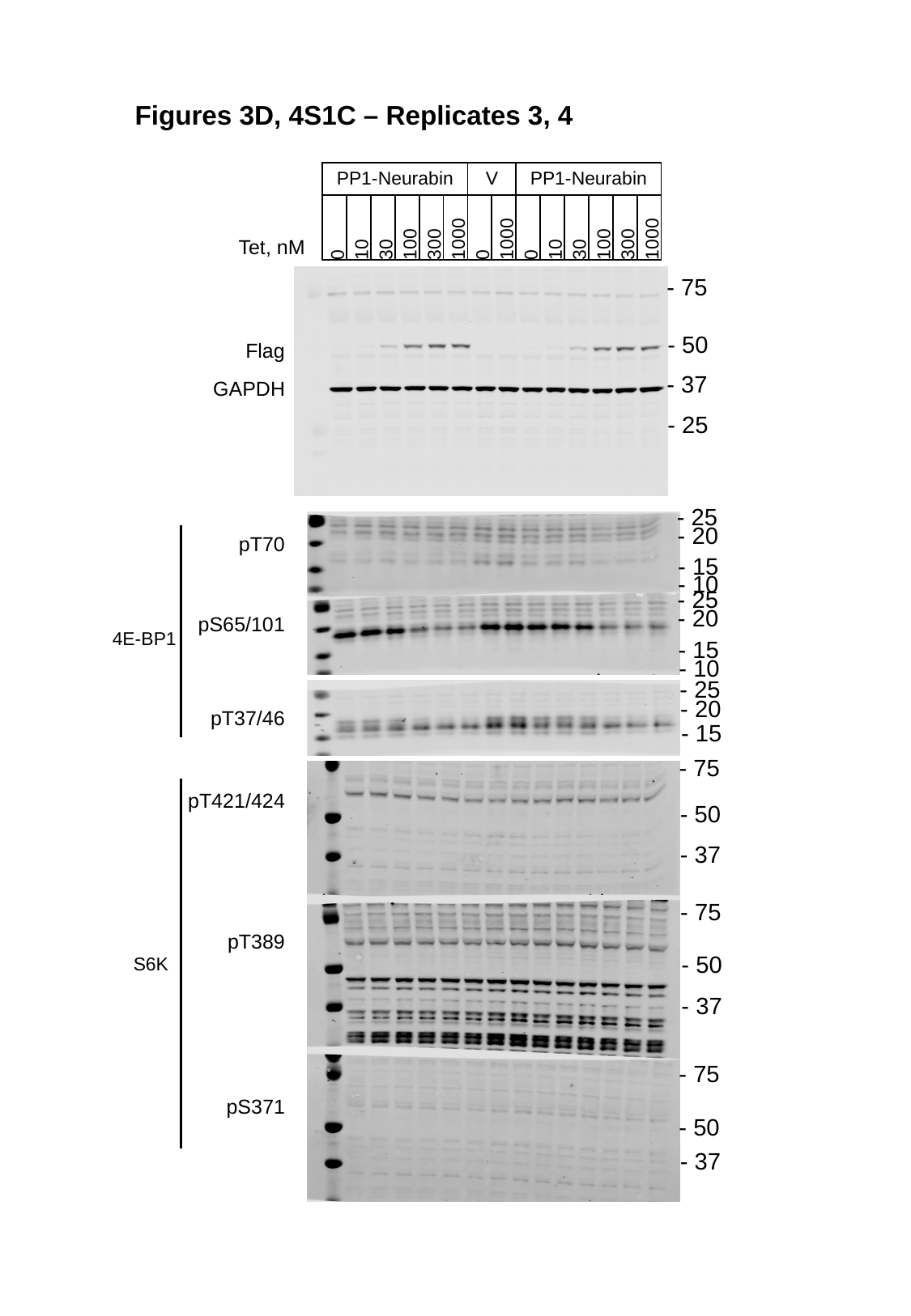

Figures 3D, 4S1C – Replicates 3, 4
| PP1-Neurabin | | | | | | V | | PP1-Neurabin | | | | | |
| --- | --- | --- | --- | --- | --- | --- | --- | --- | --- | --- | --- | --- | --- |
| 0 | 10 | 30 | 100 | 300 | 1000 | 0 | 1000 | 0 | 10 | 30 | 100 | 300 | 1000 |
Tet, nM
- 75
- 50
Flag
- 37
GAPDH
- 25
- 25
- 20
pT70
- 15
- 10
- 25
- 20
pS65/101
4E-BP1
- 15
- 10
- 25
- 20
pT37/46
- 15
- 75
pT421/424
- 50
- 37
- 75
pT389
- 50
S6K
- 37
- 75
pS371
- 50
- 37

## Slide 3
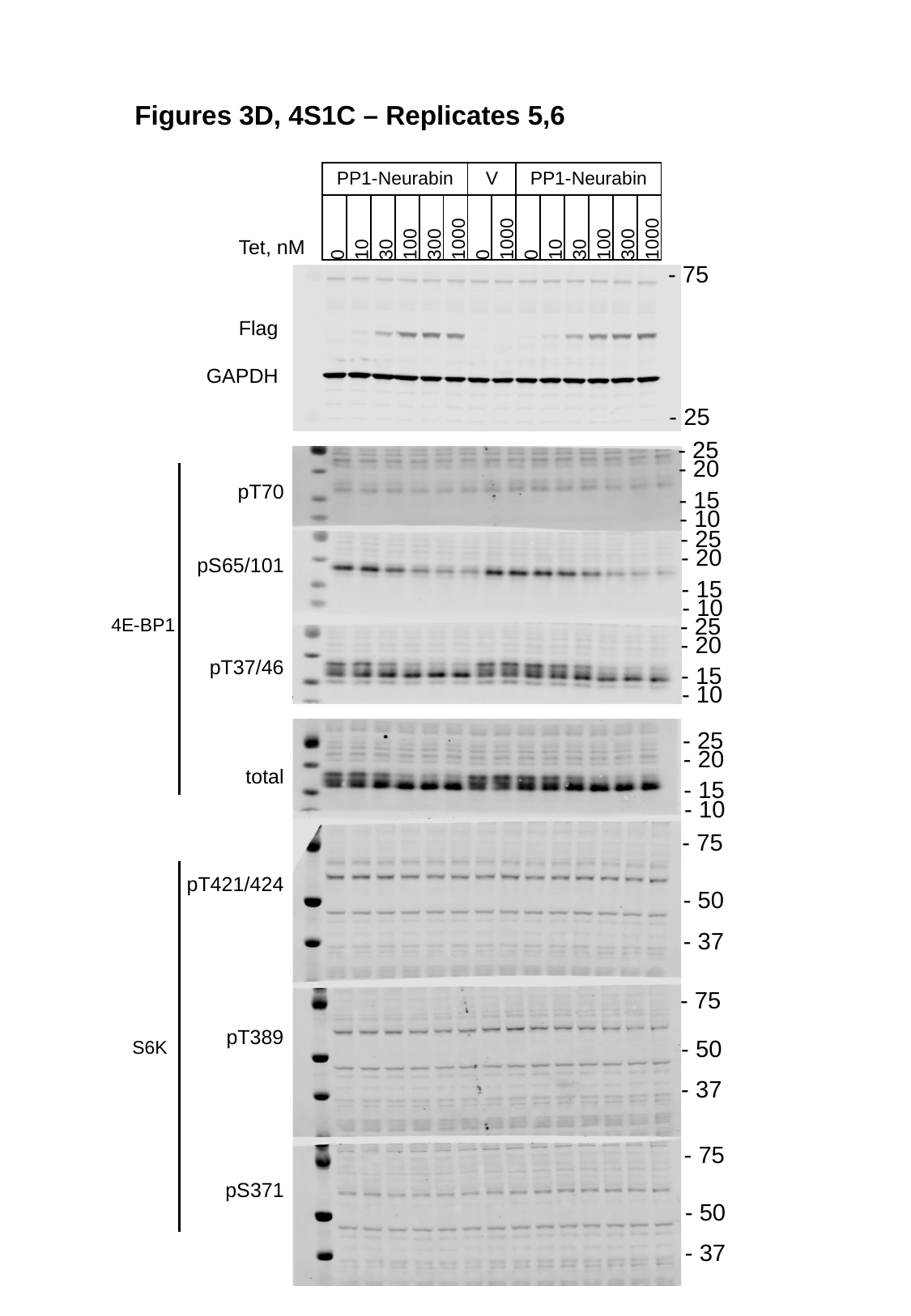

Figures 3D, 4S1C – Replicates 5,6
| PP1-Neurabin | | | | | | V | | PP1-Neurabin | | | | | |
| --- | --- | --- | --- | --- | --- | --- | --- | --- | --- | --- | --- | --- | --- |
| 0 | 10 | 30 | 100 | 300 | 1000 | 0 | 1000 | 0 | 10 | 30 | 100 | 300 | 1000 |
Tet, nM
- 75
Flag
GAPDH
- 25
- 25
- 20
pT70
- 15
- 10
- 25
- 20
pS65/101
- 15
- 10
- 25
4E-BP1
- 20
pT37/46
- 15
- 10
- 25
- 20
total
- 15
- 10
- 75
pT421/424
- 50
- 37
- 75
pT389
- 50
S6K
- 37
- 75
pS371
- 50
- 37
