# Supplementary material for: PDZ-directed substrate recruitment is the primary determinant of specific 4E-BP1 dephosphorylation by PP1-Neurabin
Source: eLife. 2025 Jun 23;13:RP103403. doi: 10.7554/eLife.103403 (PMC12185105; doi:10.7554/eLife.103403)
Supplement: Figure 3—figure supplement 1—source data 1. [file elife-103403-fig3-figsupp1-data1.zip › Figure S3B S4A.pptx]

## Slide 1
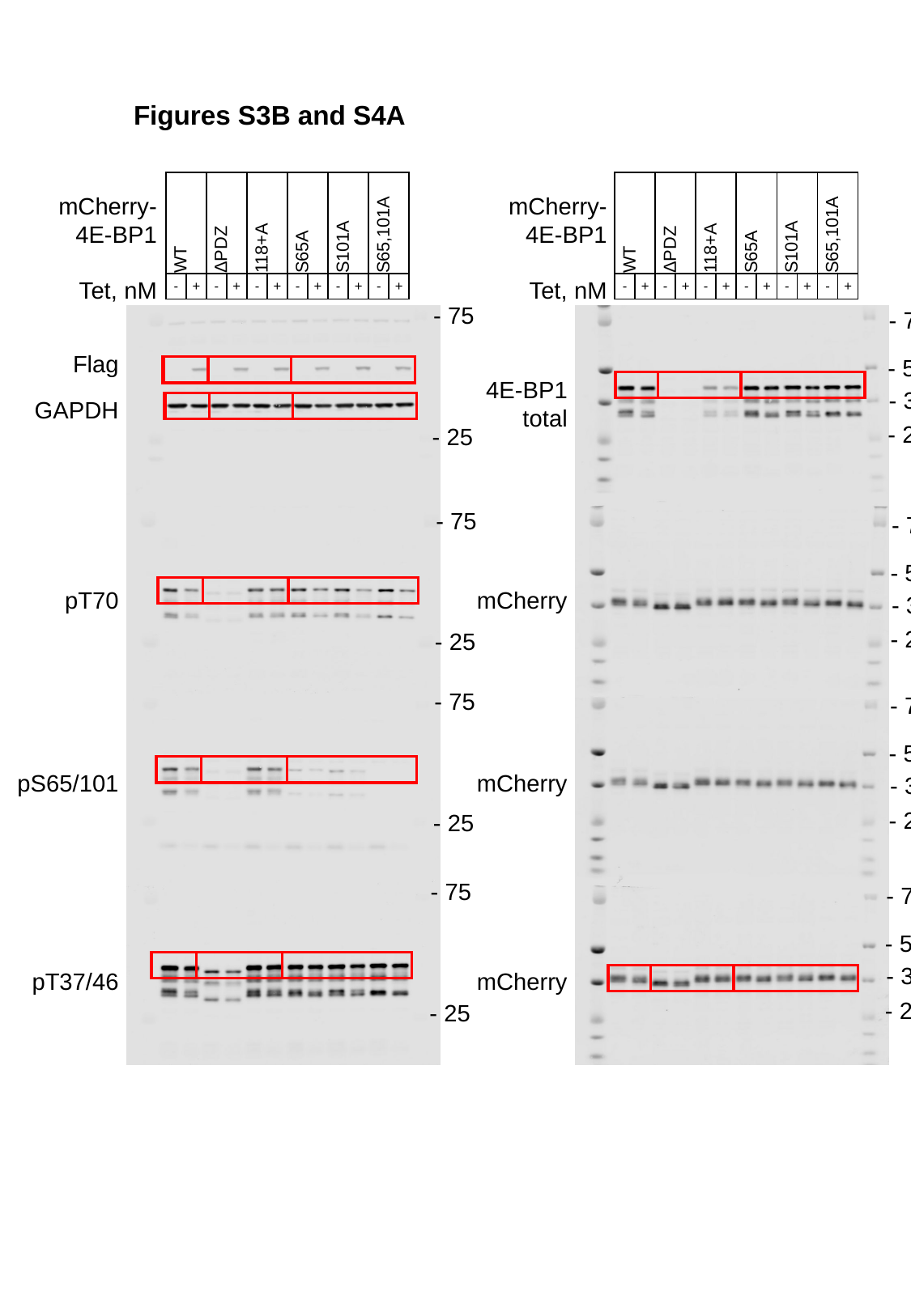

Figures S3B and S4A
| WT | | ΔPDZ | | 118+A | | S65A | | S101A | | S65,101A | |
| --- | --- | --- | --- | --- | --- | --- | --- | --- | --- | --- | --- |
| - | + | - | + | - | + | - | + | - | + | - | + |
| WT | | ΔPDZ | | 118+A | | S65A | | S101A | | S65,101A | |
| --- | --- | --- | --- | --- | --- | --- | --- | --- | --- | --- | --- |
| - | + | - | + | - | + | - | + | - | + | - | + |
mCherry-
4E-BP1
mCherry-
4E-BP1
Tet, nM
Tet, nM
- 75
- 75
Flag
- 50
4E-BP1
total
- 37
GAPDH
- 25
- 25
- 75
- 75
- 50
pT70
mCherry
- 37
- 25
- 25
- 75
- 75
- 50
pS65/101
mCherry
- 37
- 25
- 25
- 75
- 75
- 50
- 37
pT37/46
mCherry
- 25
- 25
