# Supplementary material for: PDZ-directed substrate recruitment is the primary determinant of specific 4E-BP1 dephosphorylation by PP1-Neurabin
Source: eLife. 2025 Jun 23;13:RP103403. doi: 10.7554/eLife.103403 (PMC12185105; doi:10.7554/eLife.103403)
Supplement: Figure 4—source data 1. [file elife-103403-fig4-data1.zip › Western Blots 4A.pptx]

## Slide 1
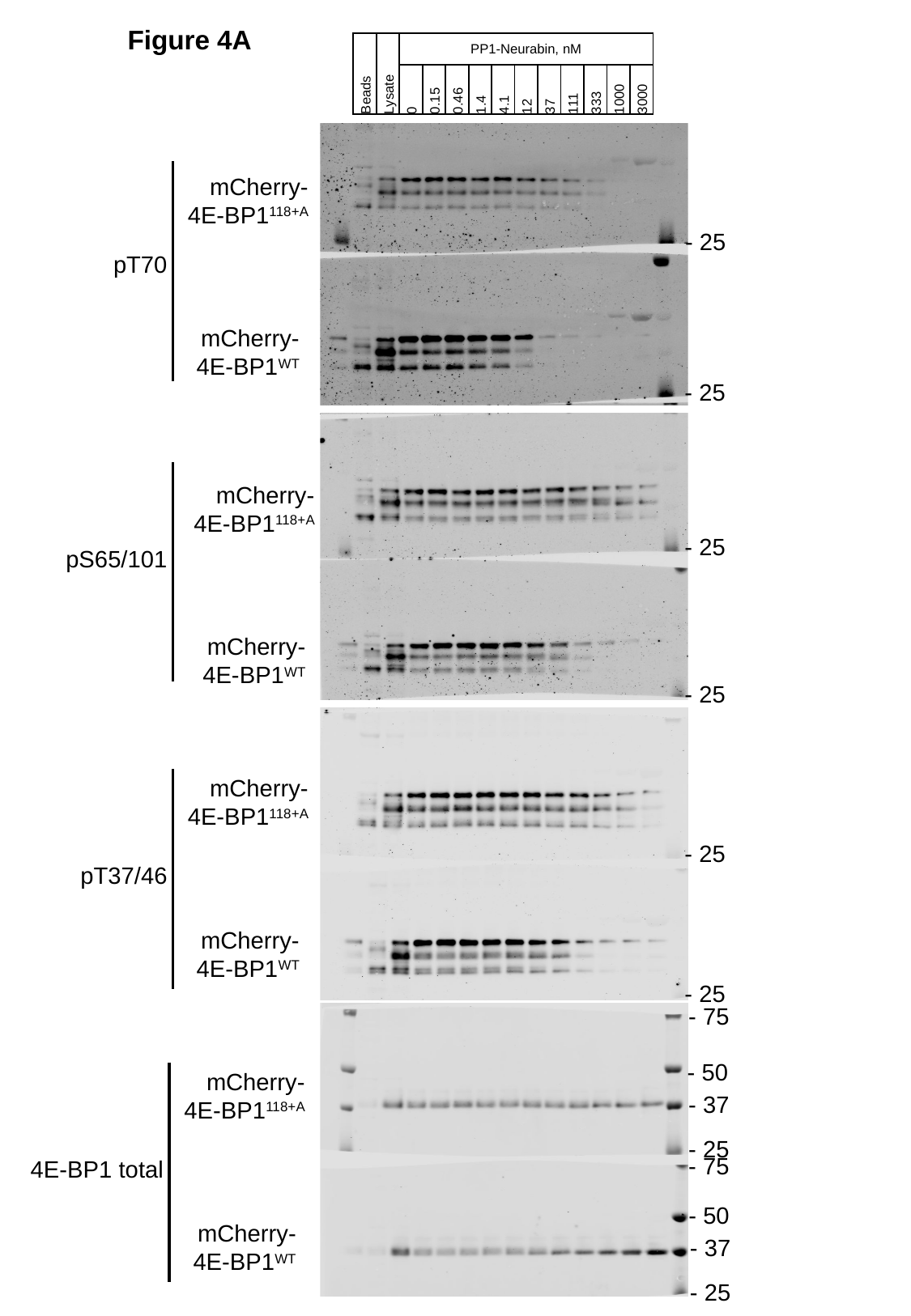

Figure 4A
| Beads | Lysate | PP1-Neurabin, nM | | | | | | | | | | |
| --- | --- | --- | --- | --- | --- | --- | --- | --- | --- | --- | --- | --- |
| | | 0 | 0.15 | 0.46 | 1.4 | 4.1 | 12 | 37 | 111 | 333 | 1000 | 3000 |
mCherry-
4E-BP1118+A
- 25
pT70
mCherry-
4E-BP1WT
- 25
mCherry-
4E-BP1118+A
- 25
pS65/101
mCherry-
4E-BP1WT
- 25
mCherry-
4E-BP1118+A
- 25
pT37/46
mCherry-
4E-BP1WT
- 25
- 75
- 50
mCherry-
4E-BP1118+A
- 37
- 25
- 75
4E-BP1 total
- 50
mCherry-
4E-BP1WT
- 37
- 25
