# Supplementary material for: PDZ-directed substrate recruitment is the primary determinant of specific 4E-BP1 dephosphorylation by PP1-Neurabin
Source: eLife. 2025 Jun 23;13:RP103403. doi: 10.7554/eLife.103403 (PMC12185105; doi:10.7554/eLife.103403)
Supplement: Figure 5—source data 1. [file elife-103403-fig5-data1.zip › raw_data_activity_assays/220512 Phosphatase assay NEB-Phactr/Protocol.docx]

1. Measured PO4 standard curve.
2. Checked activity of PP1-Phactr1 and PP1-NEB
3. THE ASSAY:

Used irsp53, irsp53-L5A and 4ebp1-70 peptides with extension including PDZ or SGS control.

Peptides were plated as 2x dilutions from 500uM – 5ul was added, 200 uM final conc. In the plate

Then 2.5 ul of 2.5 uM Phosphate sensor was added (final conc. – 0.5 uM)

As standard curve used 16uM (12.4 uM final) solution of PO4, diluted 2x.

Right before the readout, proteins were added – 2.5nM (1 nM final) PP1 or PP1-Phactr1, 0.5nM (0.2 nM final) PP1-NEB.

Readout – every 2 min, 10 points. Total volume 2.5 ul.

Layout:

Odd columns:

ABC – irsp53-PDZ

DEF – irsp53-SGS

GHI – 4ebp1-70-PDZ

JKL – PYGM15

Even columns:

ABC – 4ebp1-70-SGS

DEF – irsp53-L5A-PDZ

GHI – irsp53-L5A-SGS

JKL – PO4 std curve

Mistakes: in PP1-NEB plate added protein to PO4 std curve
