# Supplementary material for: PDZ-directed substrate recruitment is the primary determinant of specific 4E-BP1 dephosphorylation by PP1-Neurabin
Source: eLife. 2025 Jun 23;13:RP103403. doi: 10.7554/eLife.103403 (PMC12185105; doi:10.7554/eLife.103403)
Supplement: Figure 5—source data 1. [file elife-103403-fig5-data1.zip › raw_data_activity_assays/221020 PP1-NEB activity assay/Protocol 221020.docx]

1. Checked activity of 3x dilutions of from 13.5nM final PP1-Phactr1 and PP1-NEB with 100uM of IRSp53-PDZ peptide
2. THE ASSAY:

Used various peptides with PDZ or SGS C-termini.

Peptides were plated as 2x dilutions from 1000uM – 4ul was added, 400 uM final conc. In the plate

Then 2 ul of 2.5 uM Phosphate sensor was added (final conc. – 0.5 uM)

Right before the readout, 4ul of proteins were added – 1.25nM final PP1-Phactr1, 0.25 nM final PP1-NEB.

Readout – every 3 min, 6 points. Total volume 10 ul.

As standard curve used 16uM (12.4 uM final) solution of PO4, 2x dilutions. 10ul of standard plus 2.5 ul of Phosphate sensor
