# Supplementary material for: PDZ-directed substrate recruitment is the primary determinant of specific 4E-BP1 dephosphorylation by PP1-Neurabin
Source: eLife. 2025 Jun 23;13:RP103403. doi: 10.7554/eLife.103403 (PMC12185105; doi:10.7554/eLife.103403)
Supplement: Figure 6—source data 1. [file elife-103403-fig6-data1.zip › alphafold3 predictions/Legend for Supplementary Data Alphafold3.docx]

For each Alphafold3 modelling run the archive contains a .cif output file with predicted structural information. Additional files are _data.json, _summary_confidences.json and _job_request.json files that describe input and output parameters of Alphafold3 modelling.

The following folders contain Alphafold3 predictions used in this manuscript:

**fold_pp1_4ebp1_allphospho_neurabin424_594** – folder contains prediction for PP1/Neurabin/5x phosphorylated 4E-BP1 structure.

**fold_pp1_neurabin_4ebp1_pt70_chimera** – folder contains prediction for PP1-4E-BP1 chimera/Neurabin structure where 4-E-BP1 sequences are phosphorylated at T70.

**fold_pp1_neurabin_4ebp1_wt_chimera** – folder contains prediction for unphosphorylated PP1-4E-BP1 chimera/Neurabin structure.
